# How Do DICER1 Syndrome Mutations Disrupt Catalysis? Unveiling Dicer Metal Binding Architecture and Mechanism of Action Using MD Simulations and QM/MM Calculations

**DOI:** 10.1002/jcc.70446

**Published:** 2026-07-03

**Authors:** Dylan J. Nikkel, Stacey D. Wetmore

**Affiliations:** ^1^ Department of Chemistry and Biochemistry University of Lethbridge Lethbridge Alberta Canada

## Abstract

The RNA interference (RNAi) pathway regulates gene expression and viral defense and has been harnessed in therapeutic solutions to inhibit otherwise undruggable proteins by preventing translation. Dicer initiates RNAi by generating cleaved RNA products that bind to a target mRNA to promote gene silencing. Mutations to the Dicer catalytic domain cause DICER1 syndrome, which increases the risk of cancers, including early childhood variants. However, the catalytic mechanism remains poorly defined due to the lack of structural data for Dicer bound to a substrate (or substrate mimic) in the presence of divalent ions known to be critical for nuclease activity. This study uses molecular dynamics (MD) simulations to uncover the first atomic level structure of the wild‐type Dicer–RNA complex, including the binding pattern of two catalytically essential Mg^2+^ ions. Subsequently, quantum mechanics/molecular mechanics (QM/MM) techniques are used to elucidate the Dicer catalytic mechanism for phosphodiester bond cleavage. Among three fully characterized pathways, our data suggest catalysis is only feasible when both active site Mg^2+^ ions are directly coordinated to the RNA substrate and a hydroxide ion nucleophile is bound to an active site Mg^2+^ ion. Phosphodiester bond hydrolysis proceeds through a two‐step mechanism involving a phosphorane intermediate that is stabilized by direct Mg^2+^–substrate coordination and a hydrogen bond with K1806. This newly identified mechanism is consistent with experimental kinetic data, the impact of mutating the corresponding lysine in mouse Dicer, and the active site architectures and proposed mechanisms for other related nucleases. Directed by the enhanced understanding of wild‐type Dicer function, MD simulations subsequently show that six known DICER1 syndrome‐causing mutants likely impede catalysis by inducing unique active site disruptions that inhibit Mg^2+^‐ion coordination to the substrate. By furthering our knowledge of the structure and catalytic mechanism of wild‐type and mutant Dicer, this work unveils the molecular underpinnings of DICER1 syndrome and opens the door for the development of enhanced RNAi‐based therapeutics and biotechnologies.

## Graphical abstract

Dicer plays a vital role in gene regulation, making understanding its function critical for drug design and addressing human diseases. This article uses a combination of MD and QM/MM techniques to elucidate structural details of the Dicer–substrate complex, the Dicer catalytic mechanism, and the misfunction of DICER1‐syndrome‐causing mutants.

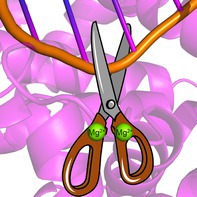

## 1 Introduction

**FIGURE 1:**
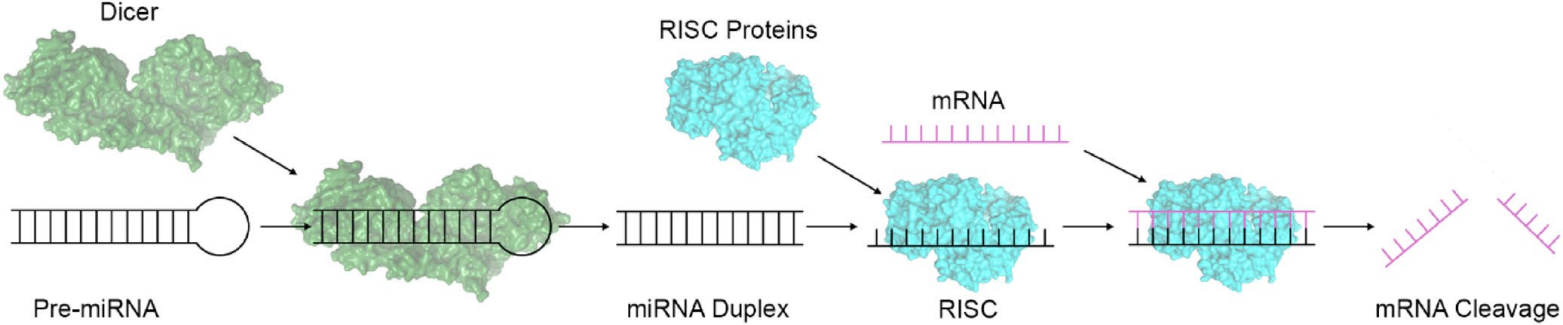
The RNA interference (RNAi) pathway in which pre‐miRNA is cleaved by Dicer to produce miRNA, which binds to the RISC to target and cleave mRNA and thereby silence genes.

RNA interference (RNAi) is a vital pathway in human cells for gene regulation and defense against viral infections (Figure 1) [1]. During RNAi, non‐coding microRNAs (miRNA) or small‐interfering RNAs (siRNA) are loaded into an Argonaute protein and associated binding partners to form the RNA induced silencing complex (RISC) [1, 2]. The RISC then binds messenger RNA (mRNA) that is complementary to the RNA guide strand and degrades the mRNA or otherwise prevents its translation [1, 2]. miRNA production is highly regulated along the entire transcription process [3, 4]. The final step of miRNA biogenesis is the cleavage of two phosphodiester bonds in the double‐stranded precursor miRNA (pre‐miRNA) by an endonuclease called Dicer, which produces the approximately 22 nucleotide long mature miRNA duplex [3, 5, 6]. Due to cell requirements of processing an extremely diverse array of miRNAs [7], Dicer has a broad substrate specificity, targeting RNA with a variety of sequences at similar rates [8, 9, 10].

miRNA has been implicated in a multitude of physiological processes in humans including cell differentiation [11], carcinogenesis [12, 13, 14], cardiovascular disease [15], the immune system [16], and viral defense [17, 18]. As a result, Dicer misfunction and miRNA dysregulation are associated with a wide variety of diseases and disorders [11, 19, 20, 21, 22, 23, 24]. Furthermore, Dicer mutations correlate with the development of lung, thyroid, and brain cancers, including early childhood variants, in a genetic disorder called DICER1 syndrome [21, 25, 26, 27, 28, 29, 30]. DICER1 syndrome arises from mutations to the DICER1 hotspot region in the catalytic domain [25, 31], with the impacted residues being proposed to bind catalytic Mg^2+^ ions that facilitate phosphodiester bond cleavage [31, 32, 33, 34]. These mutations impair the processing of miRNA‐5′ strands [31], which could compromise tumor suppressor miRNA and lead to the predisposition for cancer [22, 35, 36, 37]. Despite the direct connection between Dicer catalysis and DICER1 syndrome, the atomic details of the mechanism behind Dicer–mediated phosphodiester bond cleavage are poorly understood.

**FIGURE 2:**
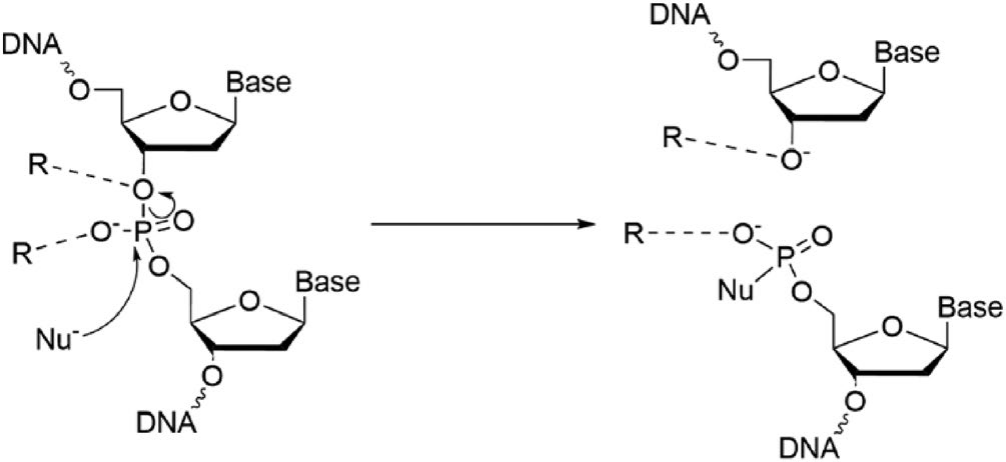
General nuclease mechanism of action. Nu represents a generic nucleophile, and R represents a metal ion (typically Mg^2+^) or protein residue.

Dicer is a large, magnesium‐dependent [34, 38], multi‐domain protein consisting of the dsRNA‐binding, DExD/H‐box helicase, DUF283, platform, PAZ, and two catalytic RNase III (labeled a and b) domains (Figure S1) [32, 33]. However, a truncated form of Dicer that only contains the RNase IIIb domain retains catalytic activity [38]. Although catalytic details are currently unclear for Dicer, nucleases function generally involves a nucleophile attacking the phosphorus atom of the RNA backbone and the enzyme stabilizing charge build up on the phosphate and leaving group (Figure 2) [39, 40]. However, the strategies different enzymes use to activate the nucleophile, stabilize intermediates, and facilitate leaving group departure vary significantly, with many endonucleases, including Dicer, utilizing metal cofactors [39, 40].

**FIGURE 3:**
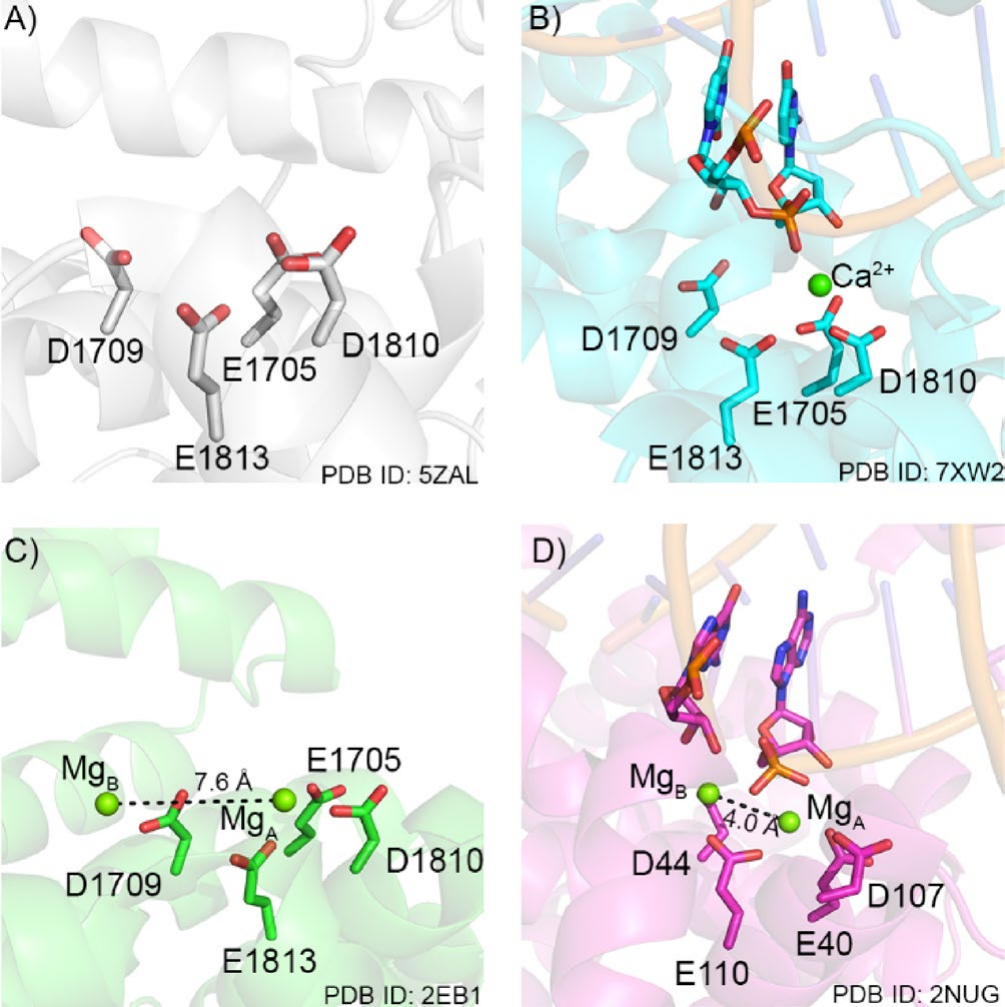
Cryo‐EM structures of the active site of (A) human Dicer without the substrate bound in the catalytic active site, and (B) Ca^2+^–inhibited human Dicer bound to RNA. X‐ray crystal structure of the active site of (C) the human Dicer RNase IIIb domain homodimer in the absence of substrate and (D) aa‐RNase III bound to substrate RNA.

Although Mg^2+^ ions are well accepted to be necessary for Dicer catalytic acivity [34, 38], the metal coordination in the active site is unclear. An early cryo‐EM structure of Dicer bound to an RNA substrate (PDB ID: 5ZAL, Figure 3A) lacks Mg^2+^ ions and the RNA is not aligned in the active site of either RNase III domain [33]. Although RNA is bound in the catalytic active site in a later cryo‐EM structure of Dicer crystallized in the presence of Ca^2+^ (PDB ID: 7XW2, Figure 3B) [32], the inhibition of enzymatic activity suggests that the active site conformation is not conducive for catalysis. A structure of the apo‐RNase IIIb domain crystallized in the presence of magnesium shows two ions bound in the active site separated by 7.6 Å in the absence of the substrate (PDB ID: 2EB1, Figure 3C) [38]. This distance is too large for both metal ions to coordinate to the substrate near the bond cleavage site as commonly observed for other nucleases including the homologous *aquifex aeolicus* (aa) RNase III (PDB ID: 2NUG, Figure 3D) [41, 42, 43, 44, 45, 46, 47, 48, 49, 50]. However, catalysis could still be possible through the observed human Dicer Mg^2+^ configuration if one metal adopts indirect (water mediated) coordination. Indeed, indirect metal coordination to the substrate leaving group occurs for many two‐metal‐dependent [45, 51, 52, 53] and one‐metal‐dependent [51, 54, 55, 56] nucleases. Nonetheless, metal location can shift upon nucleic acid substrate binding as reported for the type II restriction enzyme EcoRV [57] and proposed for Dicer [38].

In addition to questions surrounding the coordination of active site Mg^2+^ ions, the roles of active site residues are unclear. K1806 has been implicated in Dicer activity as mutation of the analogously positioned residue in mouse Dicer (K1790) inhibits catalysis (Figure S2A,B) [58]. A similarly positioned lysine residue can also be found in the active site of other nucleases (Figure S2C–F) [45, 51, 57, 58, 59, 60, 61, 62], which mutational experiments (EcoRV [57, 61] and MutH [60]) and computational studies (EndoV [51]) suggest to be important for catalysis. Nevertheless, the exact function of such a lysine in nuclease activity is currently unknown, with proposed roles including stabilizing charge build up in the transition state [51, 61], facilitating DNA bending [57], and mediating metal cofactor binding [59]. With an incomplete picture of substrate binding, Mg^2+^ ion coordination, and the roles of functionally important active site residues, questions remain regarding how Dicer cleaves phosphodiester bonds and how DICER1 syndrome‐related mutations disrupt this function.

Computational chemistry techniques have proven to be invaluable for discerning enzyme structures and catalytic mechanisms [63, 64, 65, 66, 67, 68, 69], including for nucleases [41, 47, 51, 54, 56, 70, 71, 72, 73, 74, 75, 76, 77, 78, 79, 80, 81, 82, 83, 84, 85, 86]. Specifically, molecular dynamics (MD) simulations have aided the characterization of enzyme structural features, including metal binding architectures. For example, MD simulations uncovered different apurinic/apyrimidinic endonuclease 1 (APE1)–pol‐β [87] and APE1–substrate [88, 89, 90] interactions and thereby identified residues responsible for binding active site Mg^2+^ ions and revealed clues regarding enzyme activity [91, 92, 93]. Quantum mechanics/molecular mechanics (QM/MM) calculations have also been used to elucidate the mechanisms of action for nucleases [41, 47, 51, 70, 72, 78, 79, 80, 81]. Indeed, accurate QM/MM energy barriers enable comparison to experimental data and identification of the preferred catalytic pathway among many possibilities [94]. For example, QM/MM calculations on bacterial EndoV uncovered a mechanism involving (indirect) Mg^2+^ ion−substrate coordination and a critical role of lysine as the general acid [56]. These are just a few examples of many computational studies that have provided critical knowledge about the function of nucleases [41, 47, 51, 54, 56, 70, 71, 72, 73, 74, 75, 76, 77, 78, 79, 80, 81, 82, 83, 84, 85, 86].

Due to the valuable insights into nuclease activity previously obtained from computational methods, the present study employs a combination of MD simulations and QM/MM calculations to uncover details of substrate and Mg^2+^ ion binding in the Dicer active site, the wild‐type Dicer catalytic mechanism, and the misfunction of Dicer mutants. Specifically, MD simulations were initiated from a model of the Dicer catalytic RNase IIIb domain bound to an RNA substrate to determine the relative positioning of the substrate, Mg^2+^, and key active site residues. Two models were considered that differ in the initial magnesium ion–substrate coordination based on experimental structural information (Figure 3). Subsequently, snapshots from MD simulations on each model were used to initiate QM/MM calculations to map three different pathways and identify the preferred catalytic mechanism. Armed with knowledge of the ion configuration and roles of critical active site residues, MD simulations were used to determine the impact of six DICER1 syndrome causing mutants (D1709N, D1810Y, E1705K, E1813D, E1813G, and G1809R) [25, 31] on the structural dynamics of the catalytically conducive enzyme–substrate complex. Overall, this work provides the first description of the Dicer catalytic mechanism of action, clarifies the role of a catalytic lysine that may be conserved in other endonucleases, and provides a structural rationalization for the connection between Dicer mutants and DICER1 syndrome [25, 31]. This information can be used in the future to develop innovative treatments for Dicer1 syndrome [21, 25, 26, 27, 28, 29, 30] and powerful new RNAi–based therapeutics [95, 96, 97] and biotechnologies [98, 99, 100].

## 2 Computational Methods

### 2.1 MD Simulations

**FIGURE 4:**
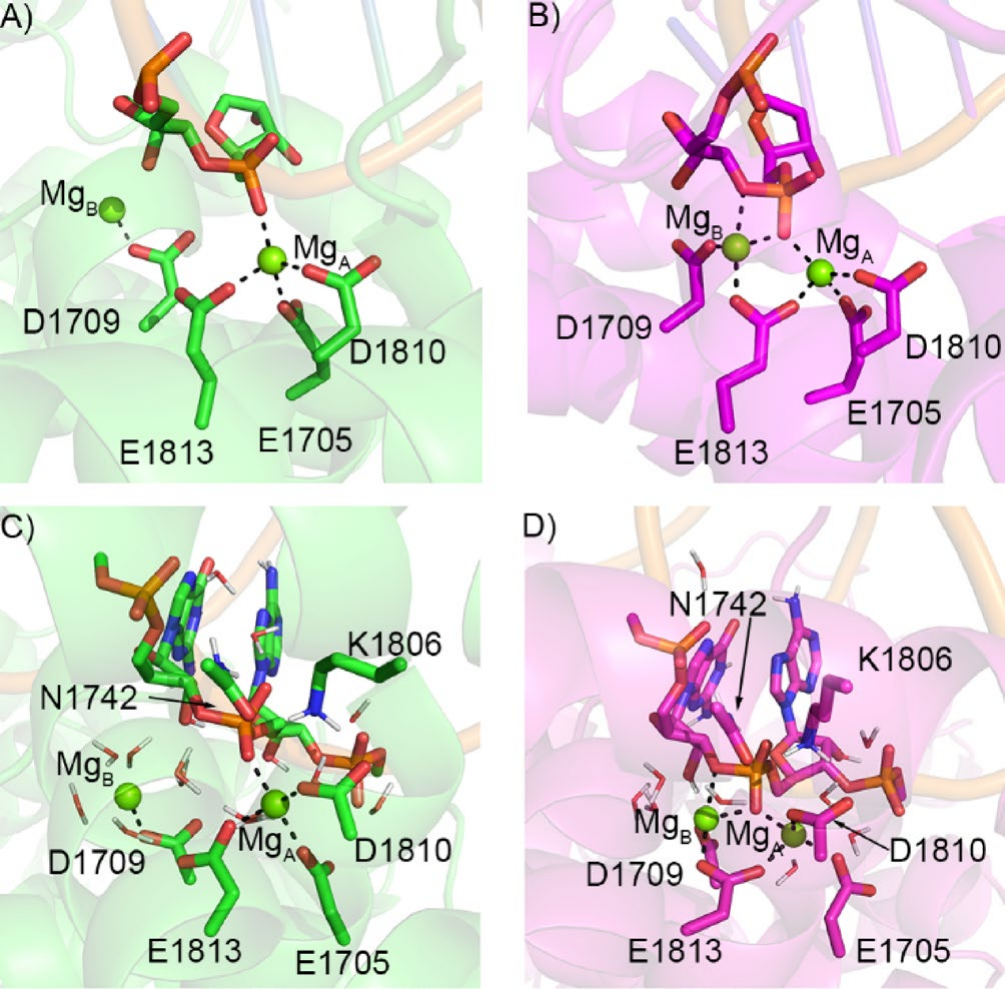
Post‐equilibration Dicer model constructed using the Mg^2+^ ion positioning from the (A) RNase IIIb homodimer (denoted RIIIb) and (B) *aa*‐RNase III (denoted aaRIII). High‐layer atoms in the QM/MM (C) RIIIb and (D) aaRIII models.

An initial model for wild‐type Dicer bound to RNA was built using the crystal structure of the Dicer RNase IIIb domain homodimer (PDB ID: 2EB1, Figure 3C) [38]. The RNase IIIb domain of Dicer and bacterial RNase III exhibit > 90% sequence identity in the active site as calculated using UniProt (Figure S3) [101]. Therefore, the substrate was added by aligning the homodimer with the crystal structure of the homologous *aa*‐RNase III bound to RNA (PDB ID: 2NUG, Figure 3D and  S4A) [49] and removing the bacterial enzyme. Side chain conformations were minimized to reduce close contacts between the RNA substrate and Dicer using PyMOL [102]. The side chain for partially resolved E142 was added using PyMOL [102]. The unresolved amino acid chain consisting of residues N122 to E141 was generated using SWISS‐MODEL [103]. Protonation states for amino acid side chains were calculated using the H++ server [104, 105]. From this starting structure, two models were generated that differ in the position and coordination geometry of the two active site Mg^2+^ ions. In the first model (denoted RIIIb), the Mg^2+^ ions are retained from the Dicer RNase IIIb homodimer crystal structure, which are separated by 7.6 Å (PDB ID: 2 EB1, Figure 4A) [38]. Specifically, Mg_A_^2+^ is coordinated to E1705, D1810, E1813, a nonbridging phosphate oxygen, and two water molecules, whereas Mg_B_^2+^ is coordinated to D1709 and 5 water molecules. In the second model (denoted aaRIII), the Mg^2+^ ions were retained from the *aa*–RNase III crystal structure and are separated by 4.0 Å (PDB ID: 2NUG, Figures 3D and 4B) [49]. Specifically, Mg_A_^2+^ has the same coordination geometry as the first model, whereas Mg_B_^2+^ is coordinated to E1813, D1709, a bridging and non‐bridging phosphate oxygen, and two water molecules.

Each model was solvated with TIP3P water molecules in a periodic rectangular prism with sides a minimum of 10 Å from the solute in each direction using the LEaP program in AMBER 2018 [106]. Sodium ions were added to neutralize the system and NaCl was added to achieve a physiological salt concentration (150 mM). The Amber force field was used throughout, with protein residues described using ff14SB parameters [107] and nucleic acid components modeled using χOL3 [108, 109].

Both the RIIIb and aaRIII models were initially minimized in a stepwise manner, using 1000 steps of steepest decent followed by 1000 steps of conjugate gradient minimization. First, the solvent was minimized, while a 100 kcal mol^−1^ Å^−2^ restraint was applied to the solute. Next, the restraint was removed from the solute hydrogen atoms. Subsequently, all solute atoms were minimized, while applying a 100 kcal mol^−1^ Å^−2^ restraint to the solvent. Finally, the entire system was minimized with no restraints, using 1000 steps of steepest decent followed by 2000 steps of conjugate gradient minimization. Each system was then heated from 10 K, with the temperature increased by 50 K every 20 ps until 310 K was reached. The restraints on the solute were subsequently dropped from 25 kcal mol^−1^ Å^−2^ to 5 kcal mol^−1^ Å^−2^ at a rate of 5 kcal mol^−1^ Å^−2^ per 20 ps. A final 20 ps equilibration step was completed while applying a solute restraint of 1.5 kcal mol^−1^ Å^−2^. For these simulations, an NVT ensemble was used, with a collision frequency of 1 ps^−1^ and a Langevin thermostat.

As an additional equilibration step for the aaRIII model, a 100 ns production simulation was performed where the distances between Mg_A_^2+^ and the phosphate non‐bridging oxygen, Mg_B_^2+^ and the phosphate non‐bridging oxygen, and Mg_B_^2+^ and O3′ were constrained to be less than 2.4 Å, which allowed the system to relax in the presence of the added Mg^2+^ ions and substrate. Following equilibration, five 500 ns MD production simulations were performed on each system. The additional equilibration step and production simulations employed an NPT ensemble with the Langevin thermostat, Berendsen barostat (1 bar), and a collision frequency of 3 ps^−1^. Minimization, equilibration, and production simulations were performed using AMBER 2018 pmemd.cuda [106]. Heavy atom RMSDs with respect to the first frame of the additional equilibration step confirm system stability (Figure S5). Overlays between the post equilibration aaRIII and RIIIb models and the Ca^2+^‐inhibited crystal structure show comparable Mg_A_^2+^−binding residues and substrate positioning (Figure S4B,C), which indicates there were no large structural shifts near the active site upon addition of the substrate and ions from the bacterial crystal structure.

Directed by calculations on the wild‐type enzyme, a wild‐type aaRIII model with a hydroxide nucleophile was generated by replacing a water bound to Mg_A_^2+^ with a hydroxide ion in the post‐equilibration aaRIII model. No additional constraints were applied to the hydroxide ion throughout the production simulations. RESP charge fitting was used to generate charges for the hydroxide ion with the R.E.D.v.III.4 program (Table S1) [110]. Similarly, D1709N, D1810Y, E1705K, E1813D, E1813G, and G1809R Dicer mutants were generated by mutating the appropriate residue using the PyMOL mutagenesis tool [102]. Each mutated side chain was manually manipulated to remove steric clashes. MD simulations were run on each system using the same protocol as outlined above. Heavy atom RMSDs with respect to the first frame of the MD production simulation show similar behavior between replicates for each mutant (Figure S6).

Trajectory analysis was conducted on the same, single active site across all replicas using the cpptraj program in AMBER 2018 [106]. Hydrogen‐bond occupancy was determine using a heavy atom distance of less than 3.0 Å and a hydrogen‐bonding angle between 135° and 180°. A residue was considered coordinated to Mg^2+^ when the distance to the metal was less than 3.0 Å. A water molecule was considered in position to attack the phosphate moiety when the distance between the phosphorus atom and water oxygen (r(O_WAT_–P)) was less than 4.2 Å and the ∠(O3′PO_WAT_) was greater than 135°. Representative structures shown in the figures were obtained by clustering using the heiragglo algorithm based on the all‐atom RMSD of the active site Mg^2+^ ions, E1705, D1709, D1810, E1813, and the nucleotide containing the phosphate to be cleaved.

### 2.2 QM/MM Methodology

QM/MM models were generated starting from MD representative structures for each wild‐type system (RIIIb and aaRIII). First, directed by the MD analysis (see results), MD simulation data were clustered (heiragglo algorithm) based on the distance between O3′ of the leaving group and Mg_B_^2+^. Since mutation of the lysine analogously positioned to K1806 in the structurally similar mouse Dicer (K1790, Figure S2B) inhibits catalysis [58], a second round of clustering based on the K1806–substrate phosphorus distance was performed to obtain the final representative structure used for QM/MM modeling. The QM region of the RIIIb model contains 172 atoms (E1705, D1709, N1742, K1806, D1810, and E1813, 2 Mg^2+^ ions, 7 metal‐coordinated waters, and 6 additional active site waters) and an overall charge of −2 (Figure 4C). The QM layer of the aaRIII model similarly has an overall charge of −2 and contains 160 atoms (E1705, D1709, N1742, K1806, D1810, and E1813 all truncated at the Cα–Cβ bond, the G and A residues 5′ and 3′ with respect to the phosphate being cleaved (truncated at C3′ and C5′, respectively), 2 Mg^2+^ ions, 4 metal‐coordinated waters, and 5 additional active site waters; Figure 4D). A model with a hydroxide nucleophile was generated from the QM/MM optimized aaRIII RC by converting one Mg^2+^–bound water into a hydroxide ion and adding an additional water molecule to the QM layer to help stabilize the more highly charged active site, resulting in a QM region with 162 atoms and a charge of −3. In all models, the MM region contained the remaining protein and substrate atoms as well as any water molecules with an atom that falls within 8 Å of any solute atom (Figure S7).

All QM/MM calculations invoked the ONIOM scheme [111]. Optimizations were performed using the M06‐2X functional [112] with the 6‐31G(d,p) basis set to treat the QM region, whereas the MM region was modeled with the same force field used for the MD simulations. This approach was chosen due its robustness and successes accurately modeling other enzymatic reactions [76, 82, 83, 113, 114, 115, 116], including metal–dependent enzymatic phosphodiester bond cleavage or formation [76, 82, 83]. Guesses for transition states (TS) were isolated from energy maxima obtained by scanning key reaction parameters (i.e., those corresponding to P–O bond formation (r(O_WAT_–P)) and cleavage (r(O3′–P))), which were subsequently subjected to unrestrained transition state optimizations. Frequency calculations were conducted to characterize the nature of all stationary points and verify that the isolated imaginary frequency was associated with the anticipated transition structure. Each pathway was verified to be continuous by mapping the internal reaction coordinate through scanning the nucleophile–electrophile and leaving group distances both to and from each stationary point. The reported reaction Gibbs energies were obtained from ONIOM(M06‐2X/6‐311+G(2df,p):AMBERff14SB) single‐point calculations. Single‐point calculations were performed with the electronic embedding (EE) scheme on structures optimized with the mechanical embedding (ME) scheme. To ensure ME optimizations do not affect the accuracy of the reaction surface, the mechanistic pathway for the aaRIII model with a water nucleophile was fully optimized with EE. Structures optimized with ME and EE are highly similar (active site all atom RMSD < 0.3 Å, Figure S8A) and the resulting energy barriers are within 11 kJ/mol (Figure S8B), supporting the robustness of the ME approach for predicting the structures of the Dicer–RNA complex. QM/MM activation barriers were compared to the estimated barrier for the catalytic step (~80–100 kJ/mol) calculated from experimental *k*_*cat*_ values (1 × 10^−1^—7 × 10^−5^ s^−1^) measured at 37°C under single and multiple turnover conditions with the pre‐let‐7a‐1 pre‐miRNA substrate [117, 118, 119].

All QM/MM calculations were performed using Gaussian 16 (Rev. B.01) [120].

### 2.3 Free Energy of Hydroxide Binding

The Gibbs energy difference for binding a hydroxide ion compared to a water molecule at Mg_A_^2+^ for the aaRIII configuration was determined using alchemical thermodynamic integration. Specifically, the endpoints were defined as the Dicer–RNA complex with the open coordination on Mg_A_^2+^ filled with waters and the Dicer–RNA complex with an Mg_A_^2+^–bound water replaced by a hydroxide ion. The aaRIII post‐equilibration structure was used as a starting point. The transition from one endpoint to the other was done in 5 steps, each consisting of a 100 ns simulation. The *λ* values for each step were 0.04691, 0.23076, 0.5, 0.76923, and 0.95308. The final energy difference was calculated using numerical integration. This same process was done using a box of 2548 water molecules, with one endpoint being a pure water box and the other endpoint having a single water replaced with OH^−^. For both systems, the simulations were repeated with the endpoints reversed. The resulting Gibbs energy values are within 0.3 kJ/mol, showing negligible hysteresis. Block averaging was used to estimate the error in the energy. Simulations were run using the same parameters and programs as the MD simulations.

## 3 Results and Discussion

### 3.1 Indirect Coordination of Mg_B_^2^ ^+^ to the Substrate Does Not Yield Sufficient Charge Stabilization of the Leaving Group to Permit Catalysis

With only a single crystal structure of Dicer containing Mg^2+^ ions bound in the active site (PDB ID: 2 EB1, Figure 3C) [38], the feasibility of the Mg^2+^ ion configuration presented in this crystal structure upon substrate binding was considered. However, the large Mg^2+^ separation prevents both ions from directly coordinating to the substrate, with only Mg_A_^2+^ being positioned for direct coordination with the scissile phosphate backbone in the Dicer−substrate complex. Nevertheless, other two‐metal‐dependent endonucleases are active with indirect Mg_B_^2+^−leaving group coordination [45, 51, 52, 53]. Furthermore, single‐metal‐dependent nucleases have been shown to use indirect metal–substrate coordination to cleave the phosphodiester bond [51, 54, 55, 56]. For example, a Mg^2+^ ion indirectly coordinates to the substrate in APE1 to facilitate catalysis through the donation of a metal−bound water proton to the O3′ leaving group [54, 77]. Therefore, a model was constructed that maintains the RIIIb ion configuration to explore indirect Mg_B_^2+^−substrate coordination in the Dicer active site.

**FIGURE 5:**
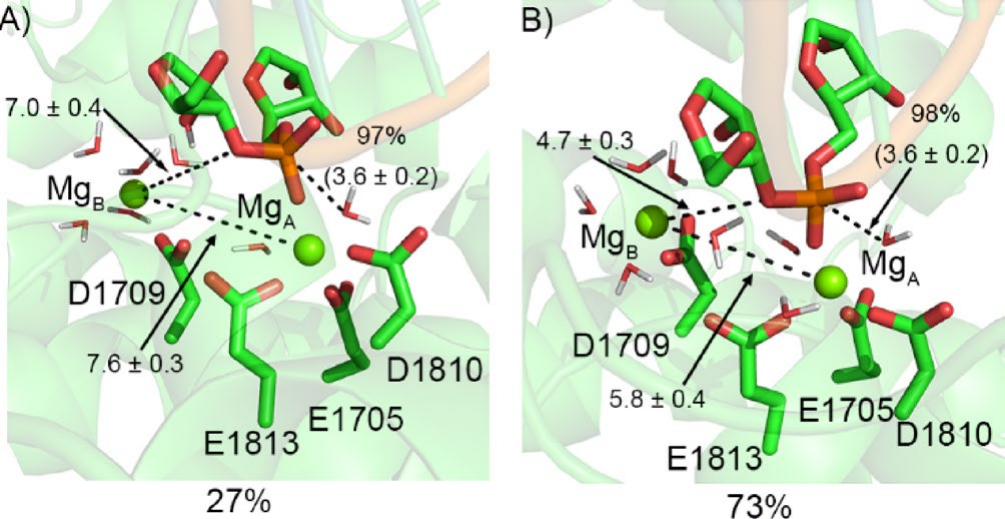
MD representative structures of the (A) Mg_B_^2+^−NC and (B) Mg_B_^2+^−IC conformations of the Dicer RIIIb active site. Occupancies over the course of the simulation provided (below). Distances are reported in Å.

Throughout MD simulations on the RIIIb model, Mg_A_^2+^ remained coordinated to E1705, D1810, E1813, a non‐bridging phosphate oxygen of the substrate, and two water molecules, whereas Mg_B_^2+^ is coordinated to D1709 and five water molecules (Figure 5). Furthermore, Mg_A_^2+^ consistently positioned a nucleophilic water for attack at the phosphate moiety (r(O_WAT_–P) = 3.6 ± 0.2 Å for 97% of the simulations time). However, two dominant active site conformations emerged that primarily differ in the distance between Mg_B_^2+^ and O3′ of the scissile phosphate, and the orientation of the Mg_B_^2+^–coordinated D1709 (∠(OδCγCβCα), Figure S9). The first, lower occupancy conformation has a long Mg_B_^2+^−substrate distance (r(Mg_B_^2+^–O3′) = 7.0 ± 0.4 Å; 27% occupancy; Figure 5A) and a Mg_B_^2+^–bound water is positioned to stabilize the substrate for less than 1% of the time in this conformation, suggesting this active site architecture is not conducive for catalysis (denoted Mg_B_^2+^−NC conformation). The second, higher occupancy conformation positions Mg_B_^2+^ closer to the substrate (r(Mg_B_^2+^–O3′) = 4.7 ± 0.3 Å; 73% occupancy; Figure 5B). Although direct Mg_B_^2+^–substrate coordination remains absent, this metal binding arrangement permits water to bridge the metal and substrate to afford indirect metal–substrate coordination as reported for other nucleases (denoted Mg_B_^2+^−IC conformation) [51, 54, 56, 76]. Indeed, a Mg_B_^2+^–coordinated water is within 3.0 Å of O3′ for 100% of the time this conformer is adopted, which could potentially protonate the leaving group during departure as seen for APE1 [54]. Interestingly, a K1806–substrate hydrogen bond rarely forms during the simulations regardless of the Mg_B_^2+^ positioning (occupancy < 2%), which raises questions regarding the viability of this metal binding architecture since mutation of the corresponding residue in mouse Dicer significantly impacts catalysis [58].

**FIGURE 6:**
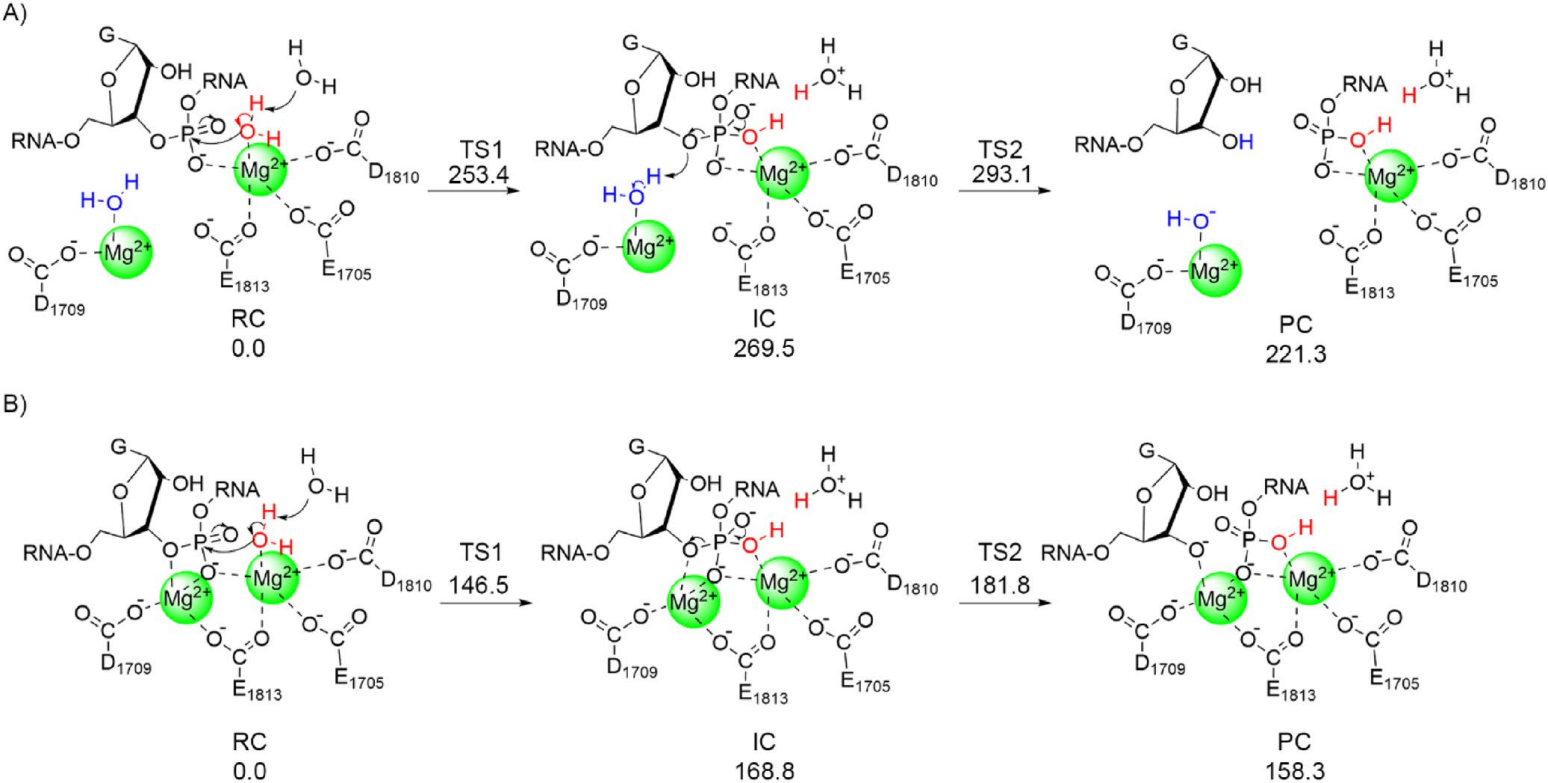
ONIOM(M06‐2X/6‐311+G(2df,p):AMBERff14SB)//ONIOM(M06‐2X/6‐31G(d,p):AMBERff14SB) calculated Dicer mechanism of action involving a water nucleophile for the (A) RIIIb and (B) aaRIII models. Relative energies reported in kJ/mol. Water molecules coordinated to Mg^2+^ ions that do not participate in the reaction are omitted for clarity (see Figure S10 for detailed coordination). See Figures S8 and S11 for detailed structural information.

To further probe the catalytic mechanism associated with the RIIIb model, a QM/MM RC was built from a representative MD snapshot in the Mg_B_^2+^−IC conformation. The optimized RC retains the Mg^2+^ coordination observed in the MD simulations, including indirect coordination between Mg_B_^2+^ and O3′ of the substrate (Figure 6A and Figures S10A and S11). The water nucleophile bound to Mg_A_^2+^ is positioned to attack phosphorus (r(O_WAT_–P) = 3.392 Å). The first transition state is late, with water far into nucleophilic attack (r(O_WAT_–P) = 1.963 Å) and a proton on the nucleophilic water being transferred to an unbound water (r(O_WAT_–H_WAT_) = 1.771 Å). The resulting hydronium ion is stabilized through direct hydrogen bonds to the phosphate backbone and E1705. The first TS leads to an intermediate complex, with large leaving group (1.820 Å) and nucleophilic water–phosphate (1.795 Å) distances. In the second transition state, the nucleophile–phosphate bond is almost formed (O_WAT_–P distance = 1.684 Å) and the leaving group–phosphate bond significantly extended (2.404 Å). Additionally, O3′ of the substrate has partially abstracted a proton from a Mg^2+^–coordinated water (r(O3′–H_WAT_) = 1.399 Å), which helps stabilize the charge on the leaving group. In the PC, the proton from the Mg_B_^2+^–bound water is fully transferred to the substrate, the nucleophile–phosphate bond formed, and the leaving group–phosphate bond cleaved.

Overall, the rate‐limiting barrier for this mechanism is extremely high (293.1 kJ/mol, Figure 6A and Table S2), falling ~200 kJ/mol above the estimated experimental barrier (80–100 kJ/mol) [117, 118, 119]. When coupled with the low occupancy of the K1806–substrate hydrogen bond during the MD simulations, a Dicer catalytic mechanism involving indirect Mg_B_^2+^ coordination to the substrate is unlikely. Thus, despite the predicted catalytic mechanism for other endonucleases involving similar indirect metal–substrate coordination [51, 54, 56, 76], this metal binding geometry in the RNase IIIb domain of Dicer is not conducive for catalysis.

### 3.2 Dicer Phosphodiester Bond Cleavage Facilitated by Direct Mg_B_^2^ ^+^–Substrate Coordination and a Mg_A_^2^ ^+^–Bound Water Nucleophile Is Kinetically Unfavoured

The energetic infeasibility of the Dicer pathway involving indirect metal–substrate coordination suggests insufficient O3′–leaving group stabilization is provided by the water bridging the metal and substrate. Therefore, a model was considered in which both Mg^2+^ ions are directly coordinated to the substrate, which mirrors the coordination geometry observed in the homologous *aa*‐RNase III (PBD ID: 2NUG, Figure 3D) [49]. Specifically, Mg_A_^2+^ is coordinated to E1705, D1810, E1813, a non‐bridging phosphate oxygen of the substrate, and two water molecules, while Mg_B_^2+^ is coordinated to D1709, E1813, a bridging and a non‐bridging phosphate oxygen of the substrate, and two water molecules. An analogous metal–substrate binding geometry has also been reported for other nucleases [41, 42, 43, 44, 45, 46, 47, 48, 49, 50], including EndoV [51], CRISPR‐Cas9 [70], and HIV RNase H [79].

**FIGURE 7:**
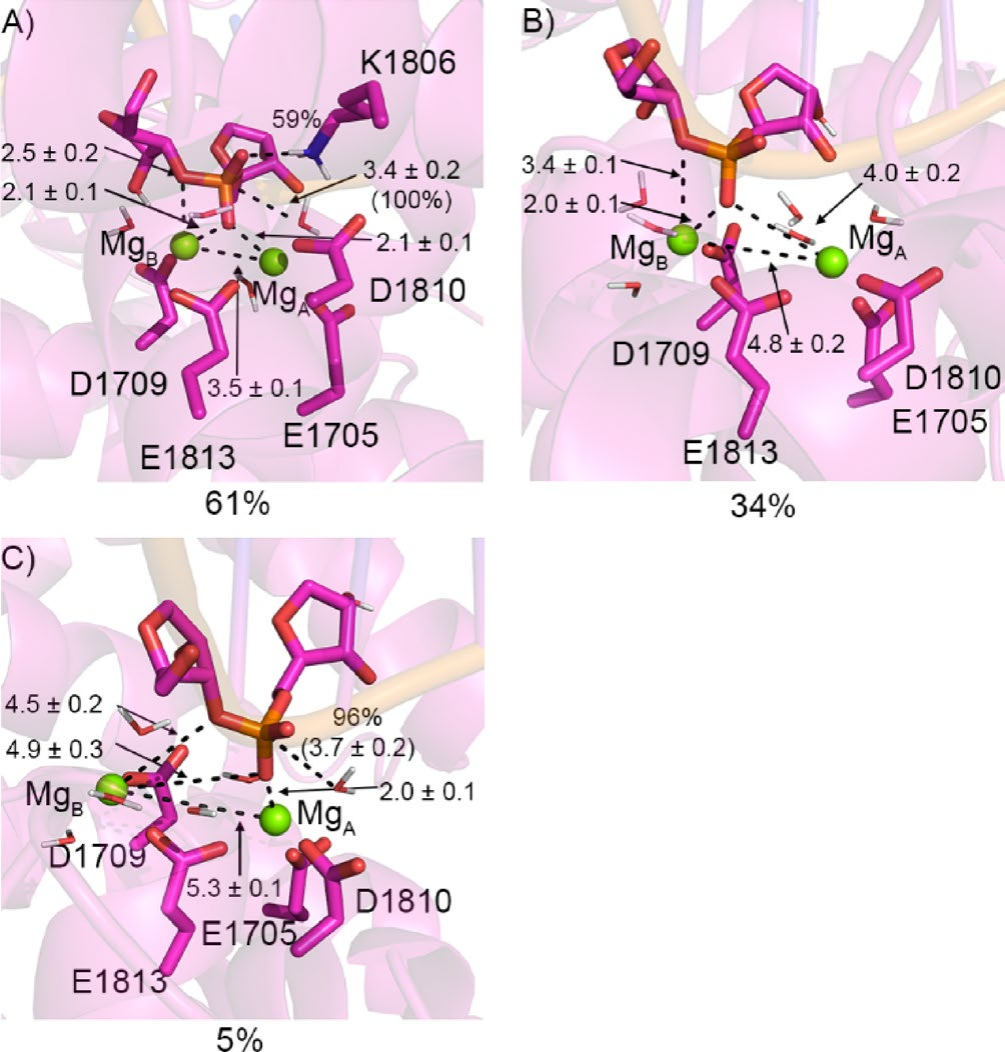
MD representative structures of the (A) 2Mg^2+^−B, (B) Mg_A_^2+^−U, and (C) Mg_B_^2+^−U conformations of the Dicer active site for the aaRIII model with a water nucleophile. Occupancies over the course of the simulation provided (below).

MD simulations highlight the conformational flexibility of the Dicer active site, with three distinct conformers adopted that differ in Mg^2+^–substrate coordination (Figure S12). For much of the MD simulation time (61%), both Mg^2+^ ions are bound to the substrate, mirroring the aaRIII structure (denoted two Mg^2+^−bound or 2 Mg^2+^−B conformation, Figure 7A). In this conformer, water is correctly positioned with respect to the substrate to initiate the reaction (100% occupancy; r(O_WAT_–P) = 3.4 ± 0.2 Å), Mg^2+^ can directly provide leaving group stabilization (r(Mg_B_^2+^–O3′) = 2.5 ± 0.2 Å), and the Mg^2+^ ions are within 3.5 ± 0.1 Å, which is comparable to the distance reported for other two‐metal‐dependent enzymes [41, 42, 43, 44, 45, 46, 47, 48, 49, 50, 86]. Additionally, K1806, which is believed to be critical for activity [58], hydrogen bonds with the substrate phosphate moiety for 59% of the simulation time. In a second metal coordination geometry (34% occupancy), Mg_A_^2+^ coordination to the non‐bridging phosphate oxygen is replaced with coordination to water (designated the Mg_A_^2+^−unbound or Mg_A_^2+^−U conformation, Figure 7B). This conformation is unlikely to be catalytically viable since a large distance between Mg_A_^2+^ and the phosphorous would prevent nucleophilic attack and the associated lengthening of the Mg_B_^2+^–O3′ coordination would significantly diminish leaving group stabilization. For the remaining simulation time (5% occupancy), direct Mg_B_^2+^ coordination to the bridging and non‐bridging phosphate oxygens is replaced by coordination to a water molecule and D1713 (denoted the Mg_B_^2+^−unbound or Mg_B_^2+^−U conformation, Figure 7C), and the K1806 hydrogen bond with the scissile phosphate is absent. While indirect Mg_B_^2+^–substrate coordination is maintained in this conformer, our previous QM/MM calculations highlight that indirect coordination is not sufficient for catalysis. Therefore, only the dominant 2Mg^2+^−B conformation has the potential to be catalytically conducive for phosphodiester bond hydrolysis.

In the QM/MM optimized RC built from the representative structure of the dominant 2Mg^2+^–B conformation, the metal ions maintain the coordination observed in the MD simulations (Figure 6B; Figures S8C and S10B). K1806 hydrogen bonds to a non‐bridging phosphate oxygen, with this interaction maintained and slightly strengthened throughout the reaction (r(O_Phosphate_–K1806) decreases by ~0.05 Å from RC to TS2). The water nucleophile is positioned to attack phosphorus (r(O_WAT_–P) = 2.911 Å). As observed for the RIIIb model, TS1 is late (r(O_WAT_–P) = 2.040 Å) and the nucleophilic water has lost a proton to generate a hydronium ion that is stabilized by direct hydrogen bonding to the phosphate backbone and E1705. The IC has long phosphorus distances to the leaving group (1.831 Å) and nucleophile (1.924 Å). The second transition state is also late (r(O3′–P) = 2.383 Å), with the nucleophile attack nearly complete (r(O_WAT_–P) = 1.717 Å). This leads to a product complex in which the backbone is cleaved and the nucleophile–phosphate bond is fully formed.

Although the rate‐limiting barrier for Dicer phosphodiester bond catalysis involving direct Mg_B_^2+^−substrate coordination (181.8 kJ/mol) is over 100 kJ/mol lower than that involving indirect Mg_B_^2+^−substrate coordination (Figure 6B), the predicted barrier remains significantly higher than the experimental estimates (~80–100 kJ/mol) [117, 118, 119]. Therefore, despite this second mechanism mirroring the catalytically favorable pathway elucidated for other two‐metal‐dependent nucleases (e.g., EndoV [51], CRISPR‐Cas9 [70], HIV RNase H [79], and BamHI [81]) this mechanism is unfeasible for Dicer.

### 3.3 Dicer Phosphodiester Bond Cleavage Is Catalytically Favorable With Direct Mg_B_^2^ ^+^–Substrate Coordination and a Hydroxide Nucleophile

Although the Dicer mechanism involving a water nucleophile that parallels that used by many two‐metal‐dependent nucleases [47, 51, 70, 78, 79, 81, 82] is associated with a high energy barrier, hydroxide ions are also available in a cellular environment and are much stronger nucleophiles. Albeit existing at a much lower concentration than water, a hydroxide nucleophile is known to be utilized by other two‐metal‐dependent nucleases [41, 81, 84, 85, 86, 121, 122], including cases where a water nucleophile is insufficient for catalysis [81, 84, 86]. Additionally, some hydroxide–dependent nucleases, such as Ribonuclease H [41], BamHI [81], and HIV‐1 [84], have similar active site architectures to Dicer, with 4 D/E residues binding two Mg^2+^ ions in the active site. To determine whether a hydroxide ion could be generated in the active site by deprotonating a Mg^2+^‐bound water, QM/MM calculations were initiated to model a water‐mediated proton transfer to the phosphate backbone of RNA or D1810. However, all attempts to identify a stable structure with hydroxide bound to Mg_A_^2+^ through these mechanisms were unsuccessful, suggesting that a hydroxide ion may instead bind to Mg_A_^2+^ from solution.

Thermodynamic integration was used to determine the energetic feasibility of binding a hydroxide ion in the Dicer active site as done for other enzymes (Scheme S1) [84, 123, 124], including nucleases [84, 123]. Our calculations predict that hydroxide binding is favorable, with a binding free energy of −21.7 ± 1.8 kJ/mol (Figure S13). However, the free energy penalty for binding a low concentration ligand is estimated to be 50.1 kJ/mol based on an initial volume (V_i_) calculated assuming a 1 × 10^−7^ M concentration of hydroxide in solution and a water density of 0.997 g/mL, the final volume (V_f_) calculated based on two potential hydroxide binding sites on Mg_A_^2+^, and a temperature of 310 K (Equation S1). Overall, the total energy cost for hydroxide binding (28.4 kJ/mol) is significantly less than the experimental barrier for Dicer catalysis (80–100 kJ/mol) [117, 118, 119], suggesting that binding of a nucleophilic hydroxide ion to Mg_A_^2+^ in the Dicer active site is energetically viable.

**FIGURE 8:**
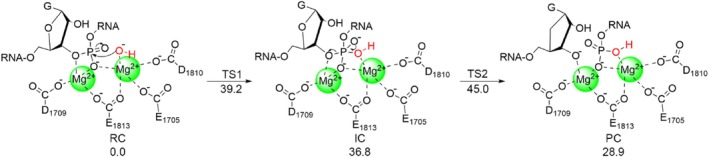
ONIOM(M06‐2X/6‐311+G(2df,p):AMBERff14SB)//ONIOM(M06‐2X/6‐31G(d,p):AMBERff14SB) calculated Dicer mechanism of action for the aaRIII model with a hydroxide nucleophile. Relative energies reported in kJ/mol. Water molecules coordinated to Mg^2+^ ions that do not participate in the reaction are omitted for clarity (see Figure S10 for detailed coordination). See Figure S14 for detailed structural information.

Due to charge repulsion with the substrate, the hydroxide nucleophile is positioned slightly further (~0.15 Å) from phosphorus in the QM/MM optimized RC compared to the water nucleophile (Figures S8, S10, and S14). K1806 hydrogen bonds with a non‐bridging phosphate oxygen, which strengthens as the reaction progresses (hydrogen‐bond distance decreases by ~0.13 Å). Unlike the late TS characterized with a water nucleophile, the transition state corresponding to hydroxide ion attack is early (long R(O_OH_–P) distance of 2.406 Å), which results in a barrier of 39.2 kJ/mol. The phosphorane intermediate contains a slightly extended phosphorus–hydroxide bond (1.805 Å) and an elongated phosphorus–leaving group distance (1.940 Å) and is slightly thermodynamically stable compared to TS1 (Figure 8). TS2 is also earlier than the analogous transition state involving a water nucleophile, with a long phosphorus–nucleophile distance (1.800 Å) and a short phosphorus–leaving group distance (1.967 Å). The strong nucleophile coupled with direct Mg_B_^2+^–O3′ coordination for leaving group stabilization leads to a low rate‐limiting barrier of 45.0 kJ/mol (Figure 8). When combined with the previously estimated penalty for hydroxide binding in the active site, the predicted total energetic cost for the reaction is 73.4 kJ/mol.

**FIGURE 9:**
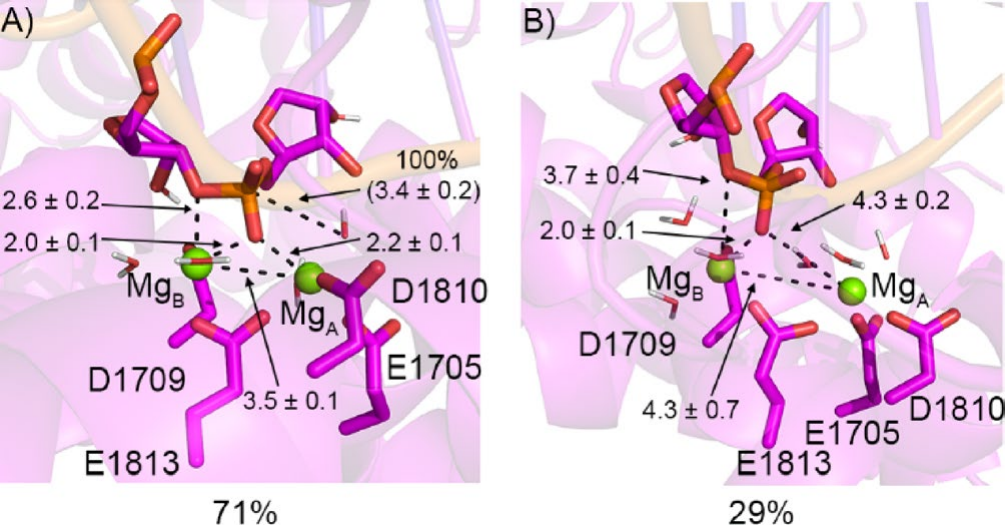
MD representative structures of the (A) 2Mg^2+^−B and (B) Mg_A_^2+^−U conformations of the Dicer active site for the aaRIII model with a hydroxide nucleophile. Occupancies over the course of the simulation provided (below).

In addition to a hydroxide nucleophile resulting in a catalytically viable barrier, MD simulations suggest a Mg_A_^2+^‐bound hydroxide does not significantly change the active site structural dynamics. Specifically, the major active site conformation (71% occupancy, Figure 9A) mirrors the 2Mg_A_^2+^–B conformation for a water nucleophile (active site RMSD = 0.682 Å, Figure S15), with Mg_A_^2+^ coordinated to E1705, D1810, E1813, a non‐bridging phosphate oxygen of the substrate, one water molecule, and the hydroxide nucleophile, whereas Mg_B_^2+^ is coordinated to D1709, E1813, a bridging and a non‐bridging phosphate oxygen of the substrate, and two water molecules. The hydroxide nucleophile is well positioned for nucleophilic attack (r(O_OH_–P) = 3.4 ± 0.2 Å; 100% occupancy), and a hydrogen bond between the substrate and K1806 is formed for 39% of the simulation time. The minor active site conformer (29% occupancy, Figure 9B) corresponds to the Mg_A_^2+^−U conformation, with Mg_A_^2+^ coordination to the non‐bridging phosphate oxygen and Mg_B_^2+^ coordination to the bridging phosphate oxygen replaced with coordination to water, and the K1806–substrate hydrogen bond absent. Thus, in addition to being energetically viable, binding of the hydroxide nucleophile in the Dicer active site does not significantly change the active site structural dynamics.

Overall, our combined MD and QM/MM approach provides the first structural description of the Dicer RNase III domain bound to the substrate RNA and Mg^2+^ in a catalytically conducive conformation and the first characterized Dicer mechanism of action that is fully consistent with existing experimental data. The preferred Dicer catalytic mechanism is predicted to involve nucleophilic attack of a Mg_A_^2+^‐bound hydroxide ion at the scissile phosphate, which breaks the bond between the phosphate and the O3′ leaving group in two steps. The calculated rate‐limiting barrier (73.4 kJ/mol) is consistent with experimental estimates (~80–100 kJ/mol) [117, 118, 119]. In the active site, both Mg^2+^ ions directly coordinate to the RNA substrate, with Mg_A_^2+^ also coordinating to E1705, D1810, E1813, a water molecule and a hydroxide ion, and Mg_B_^2+^ also coordinating to D1709, E1813, and two water molecules. This predicted catalytically active Mg^2+^ configuration (Figure 9A) differs from that found in the crystal structure of the human Dicer RNase IIIb domain in the absence of a substrate (PDB ID: 2 EB1, Figure 3C). As reported for other nucleases [54, 57, 75, 125], this suggests that the Mg^2+^ ion binding configuration in the crystal structure differs from the catalytically active state and that the Mg^2+^ ions migrate upon substrate binding. Support for the proposed mechanism also comes from the analogous two‐metal mediated mechanisms characterized for other enzymes that cleave phosphodiester bond, such as Ribonuclease H [41], BamHI [81], and HIV‐1 [84], which similarly use 4 D/E residues to bind two Mg^2+^ ions, have both Mg^2+^ ions directly coordinated to the substrate, and require a hydroxide nucleophile for activity. Furthermore, along with substrate–Mg^2+^ coordination, our calculations clarify K1806 plays a role in stabilizing the charge forming on the substrate as the reaction proceeds when the enzyme–substrate complex adopts a catalytically‐conducive conformation, which is consistent with mutations to the analogous residue in mouse Dicer inhibiting catalysis [58]. Interestingly, a lysine with unknown function is similarly positioned next to the substrate in the active sites of several endonucleases (Figure S2C−F) [126, 127, 128], which our calculations suggest may have comparable functions. However, more research is needed to determine whether the substrate−stabilizing role observed for K1806 is conserved in the broader endonuclease family.

The details obtained from the present work regarding the Dicer catalytic pathway and the roles of active site residues in catalysis will add to the molecular toolbox for the rational design of artificial nucleases. Indeed, artificial nucleases are being developed to target specific RNA sequences [129, 130, 131] and an in‐depth knowledge of the structure of the Dicer–RNA complex and the mechanism for RNA hydrolysis will be invaluable for adjusting active site composition to enhance activity. Additionally, as Dicer is involved in the first step of RNAi, understanding Dicer function has implications for drug design. Indeed, highly modified RNAi‐based therapeutics are seeing widespread development [95, 96, 97], with the goal to use Dicer to cleave siRNA therapeutics as a way to avoid immune response activation [132, 133]. The comprehensive knowledge of the structure of the Dicer–substrate complex and Dicer mechanism of action provided by this work will aid predictions of how Dicer interacts with potential siRNA therapeutics to facilitate the design of new drug with optimal performance. As discussed in the following section, understanding the function of wild‐type Dicer can also provide insights into human diseases.

### 3.4 DICER1 Syndrome Related Mutations Disrupt Catalytically Conducive Mg^2+^ Coordination of the Substrate in the Dicer Active Site

As an enzyme that plays an integral role in miRNA biogenesis, Dicer is closely involved in maintaining human health [11, 19, 20, 21, 22, 23, 24]. As such, mutations in the catalytic core of the Dicer RNase IIIb domain increase susceptibility to disease, a genetic disorder called DICER1 syndrome [21, 25, 26, 27, 28, 29, 30]. Although these mutations are known to impair miRNA processing [31], a structural rationale for the reduced enzymatic activity of DICER1‐syndrome‐causing mutants is not currently available. With a new understanding of the structure of the wild‐type Dicer–substrate complex, Dicer active site Mg^2+^ ion configuration, and Dicer catalytic mechanism, DICER1‐syndrome‐causing mutants can be investigated to shed light on the mechanism of their pathogenic outcomes.

**FIGURE 10:**
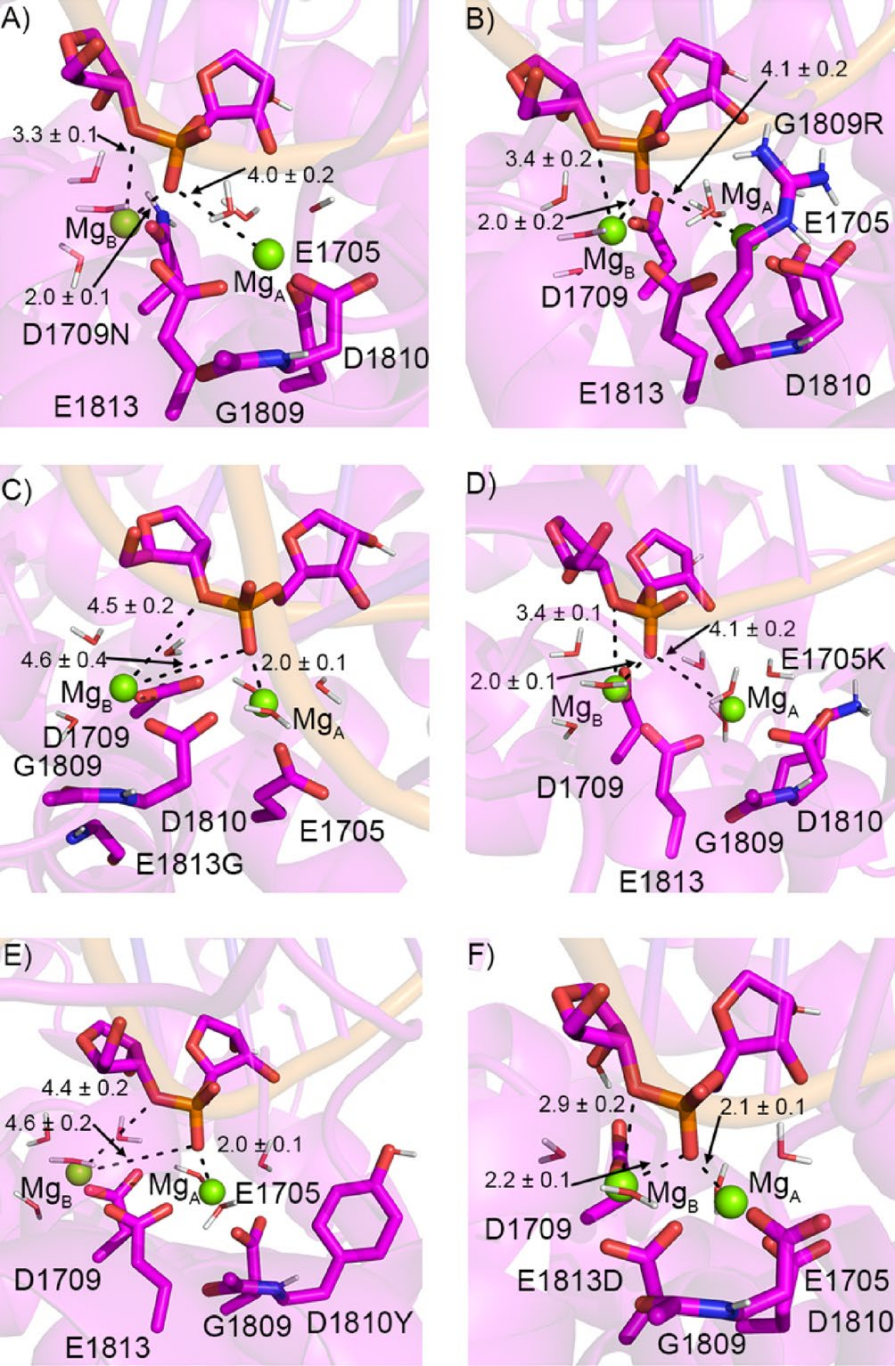
MD representative structures of dominant conformations for the (A) D1709N, (B) G1809R, (C) E1813G, (D) E1705K, (E) D1810Y, and (F) E1813D Dicer mutants. Distances are in Å, and the occupancy of a correctly positioned nucleophile is reported as a percentage.

We consider the most common D1709N, D1810Y, E1705K, E1813D, E1813G, and G1809R DICER1 syndrome mutants, which also cover all DICER1 hotspots [25]. MD simulations initiated from the catalytically–active metal configuration (aaRIII, 2Mg^2+^−B) suggest that the same Mg^2+^−binding conformations observed for wild‐type Dicer are sampled for the mutants (Table 1 and Figures 10 and S16). However, the conservation of the dominant catalytically active conformation that maintains two‐metal coordination to the substrate (2Mg^2+^−B) significantly decreases for all mutants. Furthermore, the relative persistence of the inactive conformation that involves substrate unbinding from Mg_A_^2+^ (Mg_A_^2+^−U conformation), which would render nucleophilic attack required for catalysis infeasible, and the inactive conformation involving substrate unbinding from Mg_B_^2+^ (Mg_B_^2+^−U conformation), which would decrease intermediate and leaving group stabilization required for phosphodiester bond hydrolysis, differs from the wild‐type model in a mutant‐dependent manner.

**TABLE 1:** Active site conformation occupancies for wild‐type Dicer and DICER1‐syndrome‐causing mutants observed in MD simulations^a^.

Conformation^b^	Wild‐type	D1709N	G1809R	E1813G	E1705K	D1810Y	E1813D
2Mg^2+^−B	61%–71%	6%	1%	< 1%	28%	2%	44%
Mg_A_^2+^−U	29%–34%	94%	99%	20%	52%	41%	21%
Mg_B_^2+^−U	0%–5%	0%	0%	80%	20%	57%	35%

^a^Occupancies are a percentage of frames across all replicas with a specific Mg^2+^ ion coordination (Figures 6, 9, and 10, and S16).

^b^ 2Mg^2+^−B represents the catalytically conducive conformation with both metals bound to the substrate, whereas the substrate is unbound to Mg_A_^2+^ (Mg_A_^2+^−U) or Mg_B_^2+^ (Mg_B_^2+^−U) in the other conformations.

The G1809R and D1709N mutants do not change the amino acid coordination to the Mg^2+^ ions since N1709 is able to use a side chain oxygen to coordinate to Mg_B_^2+^ and G1809 does not coordinate to a metal in the wild‐type enzyme. Nevertheless, the change in charge upon D1709N and G1809R mutation renders Mg_A_^2+^ coordination to the substrate phosphate moiety almost completely absent throughout the simulation (94%–99% Mg_A_^2+^−U, Table 1 and Figure 10A,B). In contrast, the E1813G, E1705K, and D1810Y mutants alter residues that our calculations highlight coordinate to Mg_A_^2+^, which leads to a drastic reduction in the occupancy of the 2Mg^2+^−B conformation compared to wild‐type Dicer (~1%–28%, Table 1 and Figure 10C–E). Indeed, the Mg_A_^2+^−U conformation is more prevalent for the E1705K and D1810Y mutants than wild‐type Dicer (increased to 41%–52%), which leads to less favorable nucleophile positioning (73%–86%, r(O_WAT_–P) = 3.4–3.5 Å). Furthermore, all three mutants increase the occupancy of the Mg_B_^2+^−U conformation (~20%–80%) that is rarely sampled by wild‐type Dicer (< 5%). Finally, E1813D is the only mutation considered that maintains the overall active site charge, and therefore the 2Mg^2+^−B conformation, for a significant portion of the simulation (44%, Table 1 and Figure 10F). Nevertheless, due to the shorter aspartate side chain, Mg_B_^2+^ coordination to the substrate is weakened upon E1813D mutation (average Mg_B_^2+^–O3′ distance increasing from 2.5 ± 0.2 Å for wild‐type Dicer to 2.9 ± 0.2 Å), which decreases the occupancy of 2Mg_B_^2+^−B to 44% from >61% and increases the occupancy of Mg_B_^2+^−U to 35% from < 5% for wild‐type Dicer.

Taken together, our results provide critical insight into the molecular underpinnings of DICER1 syndrome. Our QM/MM calculations on wild‐type Dicer highlight the requirement of correct coordination of the two Mg^2+^ ions in the active site for nucleophile activation and positioning, and intermediate and leaving group stabilization to yield a viable catalytic mechanism. However, all mutants investigated disrupt the preferred active site Mg^2+^ ion binding configuration, explaining their observed reduced RNA processing capabilities [134, 135, 136]. Nevertheless, the active site Mg^2+^ ion binding configuration is altered in a mutant‐dependent way by either reducing Mg_A_^2+^ coordination to the substrate (higher Mg_A_^2+^−U conformer occupancy for D1709N and G1809R), eliminating Mg_B_^2+^ coordination to the substrate (higher Mg_B_^2+^−U conformer occupancy for E1813G, E1705K, and D1810Y), or disrupting Mg_B_^2+^–leaving group stabilization (longer Mg_B_^2+^−O3′ distance for E1813D). Thus, by affording the first structural descriptions of the active site for DICER1 syndrome mutants, our simulations improve our understanding of why cells with DICER1 syndrome hotspot mutations exhibit reduced miRNA biogenesis [134, 135, 136]. This enhanced appreciation of Dicer mutant complexation with substrate RNA promises to aid the future rational design of treatments for this devastating disorder.

## 4 Conclusions

The present work uses a combined MD and QM/MM approach to provide the first structural description of the Dicer RNase III domain bound to the substrate RNA and Mg^2+^ co‐factors in a catalytically conducive conformation, and to characterize the previously unknown Dicer catalytic pathway. The preferred Dicer mechanism of action invokes a hydroxide ion bound to Mg_A_^2+^ as a nucleophile to attack the scissile phosphate, breaking the bond between the phosphate and the O3′ leaving group. The substrate is stabilized along the reaction pathway through K1806 and direct coordination to the two active site Mg^2+^ ions, which also coordinate to 4 D/E residues, water molecules and the nucleophilic hydroxide ion. Although a similar active site architecture has been reported for other nucleases, this functional wild‐type Dicer conformation differs from the Mg^2+^ ion coordination geometry in the crystal structure of the homodimer in the absence of the substrate (PDB ID: 2EB1), supporting proposals that ion migration occurs with substrate binding [38]. The calculated barrier for the reaction (73.4 kJ/mol) is consistent with experimental estimates (~80–100 kJ/mol) [117, 118, 119], and the newly proposed role for K1806 is consistent with the reduced activity of mouse Dicer upon mutation of the analogous residue [58] and the presence of a similarly positioned active site lysine in other nucleases [38, 45, 51, 57, 58, 59, 60, 61]. Although six DICER1 syndrome related mutants are found to disrupt the active site in the RNase IIIb domain in ways that would inhibit catalysis, the mutational consequences vary from disrupting Mg_A_^2+^–substrate (D1709N and G1809R) or Mg_B_^2+^–substrate (E1813G, E1705K, and D1810Y) coordination to weakening leaving group stabilization (E1813D). Regardless, these active site changes would prevent nucleophilic attack or inhibit leaving group departure, thereby providing atomic level insight into the reported reduced Dicer catalytic activity associated with DICER1 syndrome [134, 135, 136]. Overall, in addition to better understanding the chemistry of Dicer and other endonucleases with similar active site metal binding architectures and residues (lysine), the newly characterized Dicer active site structure, catalytic mechanism, and mutant function represent important steps in the development of treatments for DICER1 syndrome and other Dicer‐related disorders [11, 19, 20, 21, 22, 23, 24], and promises to accelerate the rational design of artificial nucleases [129, 130, 131] and RNAi‐based therapeutics [95, 96, 97] and biotechnologies [98, 99, 100, 132, 133].

## Funding

This work was supported by Natural Sciences and Engineering Research Council of Canada (2025‐04391), Canada Research Chairs (2021‐00484), University of Lethbridge, Alliance de recherche numérique du Canada.

## Conflicts of Interest

The authors declare no conflicts of interest.

## Supporting information


**Figure S1:** Dicer structure, highlighting the various domains.
**Figure S2:** Overlay of mouse (purple, PDB ID: 3C4B) and human (green, PDB ID: 2EB1) Dicer RNase IIIb domains highlighting (A) the overall structural similarity and (B) the similar positioning of K1806 and K1790. Crystal structures of (C) the K38A mutant EcoRV bound to DNA, (D) MutH bound to DNA, (E) Endo V, and (F) the gp2 subunit of Sf6, showing a similarly positioned active site lysine as in Dicer (K1806).
**Figure S3:** Sequence alignment of bacterial RNase III and the Dicer RNase IIIb domain. Mg^2+^‐binding regions underlined in red.
**Figure S4:** (A) Overlay of *aa*‐RNase III (magenta) and Dicer RNase IIIb homodimer (PDB ID: 2 EB1, green), highlighting key active site residues and differing locations of Mg^2+^. Overlays of the cryo‐EM structure of Ca^2+^−inhibited Dicer (cyan, PDB ID: 7XW2) and (B) the post‐equilibration aaRIII model (magenta), and (C) the post‐equilibration RIIIb model (green).
**Figure S5:** All‐heavy‐atom RMSD relative to the first frame over the MD simulations for the (A) aaRIII and (B) RIIIb wild‐type Dicer models. Five 500 ns replicates are joined back‐to‐back.
**Figure S6:** All‐heavy‐atom RMSD relative to the first frame over the MD simulations for the (A) D1709N, (B) D1810Y, (C) E1813D, (D) E1705K, (E) E1813G, and (F) G1809R Dicer mutants. Five 500 ns replicates are joined back‐to‐back.
**Figure S7:** QM/MM RIIIb (left) and aaRIII (right) models used to calculate the Dicer catalytic pathway. QM region highlighted in blue.
**Figure S8:** (A) Overlay of key ONIOM(M06‐2X/6‐311+G(2df,p):AMBERff14SB)//ONIOM(M06‐2X/6‐31G(d,p):AMBERff14SB) stationary points for the Dicer catalytic mechanism obtained using the aaRIII model employing the mechanical (magenta) and electronic (cyan) embedding schemes during geometry optimization. Calculated Dicer mechanism using (B) electronic embedding and (C) mechanical embedding during optimizations for the aaRIII model. Distances and RMSDs are reported in Å. Relative energies are reported in kJ/mol.
**Figure S9:** Histogram of the fractional occupancy of the distance (Å) between Mg_B_^2+^ and the substrate O3′ leaving group, and the ∠(OδCγCβCα) dihedral angle of D1709 (°) over the MD simulations for the RIIIb model. MD representative structures of conformations can be found in Figure 5.
**Figure S10:** ONIOM(M06‐2X/6‐31G(d,p):AMBERff14SB) optimized reactant complexes, highlighting Mg_A_^2+^ (left) and Mg_B_^2+^ (right) coordination for the (A) RIIIb and (B) aaRIII models involving a water nucleophile, and (C) aaRIII model involving a hydroxide nucleophile.
**Figure S11:** ONIOM(M06‐2X/6‐311+G(2df,p):AMBERff14SB)//ONIOM(M06‐2X/6‐31G(d,p):AMBERff14SB) calculated Dicer catalytic mechanism involving a water nucleophile obtained using the RIIIb model. Distances reported in Å. Water molecules coordinated to Mg^2+^ ions that do not participate in the reaction are omitted for clarity.
**Figure S12:** Histogram of the fractional occupancies of the distance (Å) between Mg_A_^2+^ or Mg_B_^2+^ and the substrate non‐bridging phosphate oxygen over the MD simulations for the aaRIII model. MD representative structures of conformations can be found in Figure 7.
**Figure S13:** Average *∂*V/*∂λ* from thermodynamic integration at different *λ* values for the alchemical transformation of water into hydroxide in (A) Dicer and (B) bulk solvent.
**Figure S14:** ONIOM(M06‐2X/6‐311+G(2df,p):AMBERff14SB)//ONIOM(M06‐2X/6‐31G(d,p):AMBERff14SB) calculated Dicer catalytic mechanism involving a hydroxide nucleophile obtained using the aaRIII model. Distances reported in Å.
**Figure S15:** Overlay of MD representative structures of the active (2Mg^2+^−B) conformation of the Dicer aaRIII model containing a water (magenta) and hydroxide (white) nucleophile coordinated to Mg_A_^2+^. RMSD is reported in Å.
**Figure S16:** MD representative structures for the 2Mg^2+^−B (left), Mg_A_^2+^−U (middle), and Mg_B_^2+^−U (right) conformations for the (A) D1709N, (B) G1809R, (C) E1813G, (D) E1705K, (E) D1810Y, and (F) E1813D Dicer mutants.
**Table S1:** RESP charges for a hydroxide ion calculated at the HF/6‐31G(d) level of theory.
**Table S2:** Relative energies of key stationary points for the RIIIb and aaRIII Dicer models.
**Scheme S1**. Thermodynamic cycle used to calculate the ΔΔ*G*_Binding_ for replacing water with hydroxide in the Dicer active site.
**Equation S1**.

## Data Availability

The data that support the findings of this study are openly available in Zenodo at https://zenodo.org/records/18806121, reference number DOI: 10.5281/zenodo.18806121.

## References

[1] H.‐o. Iwakawa and Y. Tomari , “Life of RISC: Formation, Action, and Degradation of RNA‐Induced Silencing Complex,” Molecular Cell 82, no. 1 (2022): 30–43, 10.1016/j.molcel.2021.11.026. 34942118

[2] S. M. Hammond , E. Bernstein , D. Beach , and G. J. Hannon , “An RNA‐Directed Nuclease Mediates Post‐Transcriptional Gene Silencing in Drosophila Cells,” Nature 404, no. 6775 (2000): 293–296, 10.1038/35005107. 10749213

[3] T. Yoshida , Y. Asano , and K. Ui‐Tei , “Modulation of microRNA Processing by Dicer via Its Associated dsRNA Binding Proteins,” Non‐Coding RNA 7, no. 3 (2021): 57, 10.3390/ncrna7030057. 34564319 PMC8482068

[4] M. Ha and V. N. Kim , “Regulation of microRNA Biogenesis,” Nature Reviews. Molecular Cell Biology 15, no. 8 (2014): 509–524, 10.1038/nrm3838. 25027649

[5] D. Zapletal , K. Kubicek , P. Svoboda , and R. Stefl , “Dicer Structure and Function: Conserved and Evolving Features,” EMBO Reports 24, no. 7 (2023): e57215, 10.15252/embr.202357215. 37310138 PMC10328071

[6] V. Ambros , B. Bartel , D. P. Bartel , et al., “A Uniform System for microRNA Annotation,” RNA 9, no. 3 (2003): 277–279, 10.1261/rna.2183803. 12592000 PMC1370393

[7] A. Kozomara and S. Griffiths‐Jones , “miRBase: Annotating High Confidence microRNAs Using Deep Sequencing Data,” Nucleic Acids Research 42, no. D1 (2014): D68–D73, 10.1093/nar/gkt1181. 24275495 PMC3965103

[8] G. Dadhwal , H. Samy , J. Bouvette , F. El‐Azzouzi , P. Dagenais , and P. Legault , “Substrate Promiscuity of Dicer Toward Precursors of the Let‐7 Family and Their 3′‐End Modifications,” Cellular and Molecular Life Sciences 81, no. 1 (2024): 53, 10.1007/s00018-023-05090-2. 38261114 PMC10806991

[9] E. Scarfiello , J. Eichlinger , and G. Meister , “The Double‐Stranded microRNA Precursor,” Postepy Biochemii 70, no. 1 (2024): 57–61. 39016229 10.18388/pb.2021_522

[10] C. T. Le , T. D. Nguyen , and T. A. Nguyen , “Two‐Motif Model Illuminates Dicer Cleavage Preferences,” Nucleic Acids Research 52, no. 4 (2024): 1860–1877. 38167721 10.1093/nar/gkad1186 PMC10899750

[11] D. P. Bartel , “Metazoan microRNAs,” Cell 173, no. 1 (2018): 20–51, 10.1016/j.cell.2018.03.006. 29570994 PMC6091663

[12] S. Messina , “The RAS Oncogene in Brain Tumors and the Involvement of Let‐7 microRNA,” Molecular Biology Reports 51, no. 1 (2024): 531, 10.1007/s11033-024-09439-z. 38637419 PMC11026240

[13] R. Kian , S. Moradi , and S. Ghorbian , “Role of Components of microRNA Machinery in Carcinogenesis,” Experimental Oncology 40, no. 1 (2018): 2–9. 29600985

[14] B. Smolarz , A. Durczyński , H. Romanowicz , K. Szyłło , and P. Hogendorf , “miRNAs in Cancer (Review of Literature),” International Journal of Molecular Sciences 23, no. 5 (2022): 2805, 10.3390/ijms23052805. 35269947 PMC8910953

[15] Z. Wang , X. Luo , Y. Lu , and B. Yang , “miRNAs at the Heart of the Matter,” Journal of Molecular Medicine 86, no. 7 (2008): 771–783, 10.1007/s00109-008-0341-3. 18415070 PMC2480593

[16] R. P. Singh , I. Massachi , S. Manickavel , et al., “The Role of miRNA in Inflammation and Autoimmunity,” Autoimmunity Reviews 12, no. 12 (2013): 1160–1165, 10.1016/j.autrev.2013.07.003. 23860189

[17] L. M. Sedger , “microRNA Control of Interferons and Interferon Induced Anti‐Viral Activity,” Molecular Immunology 56, no. 4 (2013): 781–793, 10.1016/j.molimm.2013.07.009. 23962477

[18] H. Ying , M. Ebrahimi , M. Keivan , S. E. Khoshnam , S. Salahi , and M. Farzaneh , “miRNAs; A Novel Strategy for the Treatment of COVID‐19,” Cell Biology International 45, no. 10 (2021): 2045–2053, 10.1002/cbin.11653. 34180562 PMC8426984

[19] Y. Peng and C. M. Croce , “The Role of microRNAs in Human Cancer,” Signal Transduction and Targeted Therapy 1 (2016): 15004, 10.1038/sigtrans.2015.4. 29263891 PMC5661652

[20] S. Chiosea , E. Jelezcova , U. Chandran , et al., “Overexpression of Dicer in Precursor Lesions of Lung Adenocarcinoma,” Cancer Research 67, no. 5 (2007): 2345–2350. 17332367 10.1158/0008-5472.CAN-06-3533

[21] W. D. Foulkes , J. R. Priest , and T. F. Duchaine , “DICER1: Mutations, microRNAs and Mechanisms,” Nature Reviews. Cancer 14, no. 10 (2014): 662–672, 10.1038/nrc3802. 25176334

[22] P. S. Chen , S. C. Lin , and S. J. Tsai , “Complexity in Regulating microRNA Biogenesis in Cancer,” Experimental Biology and Medicine 245, no. 5 (2020): 395–401, 10.1177/1535370220907314. 32075432 PMC7082889

[23] K. J. Dedes , R. Natrajan , M. B. Lambros , et al., “Down‐Regulation of the miRNA Master Regulators Drosha and Dicer Is Associated With Specific Subgroups of Breast Cancer,” European Journal of Cancer 47, no. 1 (2011): 138–150, 10.1016/j.ejca.2010.08.007. 20832293

[24] S. Klein , H. Lee , S. Ghahremani , et al., “Expanding the Phenotype of Mutations in DICER1: Mosaic Missense Mutations in the RNase IIIb Domain of DICER1 Cause GLOW Syndrome,” Journal of Medical Genetics 51, no. 5 (2014): 294–302, 10.1136/jmedgenet-2013-101943. 24676357 PMC4429769

[25] L. de Kock , M. K. Wu , and W. D. Foulkes , “Ten Years of DICER1 Mutations: Provenance, Distribution, and Associated Phenotypes,” Human Mutation 40, no. 11 (2019): 1939–1953, 10.1002/humu.23877. 31342592

[26] J. C. Robertson , C. L. Jorcyk , and J. T. Oxford , “DICER1 Syndrome: DICER1 Mutations in Rare Cancers,” Cancers 10, no. 5 (2018): 143, 10.3390/cancers10050143. 29762508 PMC5977116

[27] T. Rio Frio , A. Bahubeshi , C. Kanellopoulou , et al., “DICER1 Mutations in Familial Multinodular Goiter With and Without Ovarian Sertoli‐Leydig Cell Tumors,” JAMA 305, no. 1 (2011): 68–77, 10.1001/jama.2010.1910. 21205968 PMC3406486

[28] L. A. Doros , C. T. Rossi , J. Yang , et al., “DICER1 Mutations in Childhood Cystic Nephroma and Its Relationship to DICER1‐Renal Sarcoma,” Modern Pathology 27, no. 9 (2014): 1267–1280, 10.1038/modpathol.2013.242. 24481001 PMC4117822

[29] I. Slade , C. Bacchelli , H. Davies , et al., “DICER1 Syndrome: Clarifying the Diagnosis, Clinical Features and Management Implications of a Pleiotropic Tumour Predisposition Syndrome,” Journal of Medical Genetics 48, no. 4 (2011): 273–278, 10.1136/jmg.2010.083790. 21266384

[30] K. A. Schultz , J. Yang , L. Doros , et al., “DICER1‐Pleuropulmonary Blastoma Familial Tumor Predisposition Syndrome: A Unique Constellation of Neoplastic Conditions,” AJSP: Reviews & Report 19, no. 2 (2014): 90–100, 10.1097/pcr.0000000000000027. PMC4209484 25356068

[31] J. Vedanayagam , W. K. Chatila , B. A. Aksoy , et al., “Cancer‐Associated Mutations in DICER1 RNase IIIa and IIIb Domains Exert Similar Effects on miRNA Biogenesis,” Nature Communications 10, no. 1 (2019): 3682, 10.1038/s41467-019-11610-1. PMC6695490 31417090

[32] Y.‐Y. Lee , H. Lee , H. Kim , V. N. Kim , and S.‐H. Roh , “Structure of the Human DICER–Pre‐miRNA Complex in a Dicing State,” Nature 615, no. 7951 (2023): 331–338, 10.1038/s41586-023-05723-3. 36813958

[33] Z. Liu , J. Wang , H. Cheng , et al., “Cryo‐EM Structure of Human Dicer and Its Complexes With a Pre‐miRNA Substrate,” Cell 173, no. 5 (2018): 1191–1203, 10.1016/j.cell.2018.03.080. 29706542

[34] P. Provost , D. Dishart , J. Doucet , D. Frendewey , B. Samuelsson , and O. Rådmark , “Ribonuclease Activity and RNA Binding of Recombinant Human Dicer,” EMBO Journal 21, no. 21 (2002): 5864–5874, 10.1093/emboj/cdf578. 12411504 PMC131075

[35] M. J. Murray , S. Bailey , K. L. Raby , et al., “Serum Levels of Mature microRNAs in DICER1‐Mutated Pleuropulmonary Blastoma,” Oncogene 3, no. 2 (2014): e87, 10.1038/oncsis.2014.1. PMC3940920 24513630

[36] J. Chen , Y. Wang , M. K. McMonechy , et al., “Recurrent DICER1 Hotspot Mutations in Endometrial Tumours and Their Impact on microRNA Biogenesis,” Journal of Pathology 237, no. 2 (2015): 215–225, 10.1002/path.4569. 26033159

[37] A. Jakymiw , R. S. Patel , N. Deming , et al., “Overexpression of Dicer as a Result of Reduced Let‐7 MicroRNA Levels Contributes to Increased Cell Proliferation of Oral Cancer Cells,” Genes, Chromosomes & Cancer 49, no. 6 (2010): 549–559, 10.1002/gcc.20765. 20232482 PMC2859695

[38] D. Takeshita , S. Zenno , W. C. Lee , K. Nagata , K. Saigo , and M. Tanokura , “Homodimeric Structure and Double‐Stranded RNA Cleavage Activity of the C‐Terminal RNase III Domain of Human Dicer,” Journal of Molecular Biology 374, no. 1 (2007): 106–120, 10.1016/j.jmb.2007.08.069. 17920623

[39] W. Yang , “Nucleases: Diversity of Structure, Function and Mechanism,” Quarterly Reviews of Biophysics 44, no. 1 (2011): 1–93, 10.1017/s0033583510000181. 20854710 PMC6320257

[40] D. J. Nikkel , R. Kaur , and S. D. Wetmore , “How Can One Metal Power Nucleic Acid Phosphodiester Bond Cleavage by a Nuclease? Multiscale Computational Studies Highlight a Diverse Mechanistic Landscape,” Journal of Physical Chemistry. B 129, no. 1 (2025): 3–18, 10.1021/acs.jpcb.4c05875. 39720842

[41] M. De Vivo , M. Dal Peraro , and M. L. Klein , “Phosphodiester Cleavage in Ribonuclease H Occurs via an Associative Two‐Metal‐Aided Catalytic Mechanism,” Journal of the American Chemical Society 130, no. 33 (2008): 10955–10962, 10.1021/ja8005786. 18662000 PMC2745632

[42] C. Frazão , C. E. McVey , M. Amblar , et al., “Unravelling the Dynamics of RNA Degradation by Ribonuclease II and Its RNA‐Bound Complex,” Nature 443, no. 7107 (2006): 110–114, 10.1038/nature05080. 16957732

[43] L. S. Beese and T. A. Steitz , “Structural Basis for the 3′‐5′ Exonuclease Activity of *Escherichia coli* DNA Polymerase I: A Two Metal Ion Mechanism,” EMBO Journal 10, no. 1 (1991): 25–33, 10.1002/j.1460-2075.1991.tb07917.x. 1989886 PMC452607

[44] W. Yang and T. A. Steitz , “Recombining the Structures of HIV Integrase, RuvC and RNase H,” Structure 3, no. 2 (1995): 131–134, 10.1016/S0969-2126(01)00142-3. 7735828

[45] J. Y. Lee , J. Chang , N. Joseph , R. Ghirlando , D. N. Rao , and W. Yang , “MutH Complexed With Hemi‐ and Unmethylated DNAs: Coupling Base Recognition and DNA Cleavage,” Molecular Cell 20, no. 1 (2005): 155–166, 10.1016/j.molcel.2005.08.019. 16209953

[46] G. Palermo , A. Cavalli , M. L. Klein , M. Alfonso‐Prieto , M. Dal Peraro , and M. De Vivo , “Catalytic Metal Ions and Enzymatic Processing of DNA and RNA,” Accounts of Chemical Research 48, no. 2 (2015): 220–228, 10.1021/ar500314j. 25590654

[47] E. Rosta , M. Nowotny , W. Yang , and G. Hummer , “Catalytic Mechanism of RNA Backbone Cleavage by Ribonuclease H From Quantum Mechanics/Molecular Mechanics Simulations,” Journal of the American Chemical Society 133, no. 23 (2011): 8934–8941, 10.1021/ja200173a. 21539371 PMC3110985

[48] W. Yang , J. Y. Lee , and M. Nowotny , “Making and Breaking Nucleic Acids: Two‐Mg^2+^‐Ion Catalysis and Substrate Specificity,” Molecular Cell 22, no. 1 (2006): 5–13, 10.1016/j.molcel.2006.03.013. 16600865

[49] J. Gan , G. Shaw , J. E. Tropea , D. S. Waugh , D. L. Court , and X. Ji , “A Stepwise Model for Double‐Stranded RNA Processing by Ribonuclease III,” Molecular Microbiology 67, no. 1 (2008): 143–154. 18047582 10.1111/j.1365-2958.2007.06032.x

[50] T. A. Steitz and J. A. Steitz , “A General Two‐Metal‐Ion Mechanism for Catalytic RNA,” Proceedings of the National Academy of Sciences of the United States of America 90, no. 14 (1993): 6498–6502, 10.1073/pnas.90.14.6498. 8341661 PMC46959

[51] R. Kaur , D. J. Nikkel , and S. D. Wetmore , “Mechanism of Nucleic Acid Phosphodiester Bond Cleavage by Human Endonuclease V: MD and QM/MM Calculations Reveal a Versatile Metal Dependence,” Journal of Physical Chemistry. B 128, no. 39 (2024): 9455–9469, 10.1021/acs.jpcb.4c05846. 39359137

[52] H. Viadiu and A. K. Aggarwal , “The Role of Metals in Catalysis by the Restriction Endonuclease Bam HI,” Nature Structural & Molecular Biology 5, no. 10 (1998): 910–916. 10.1038/2352 9783752

[53] M. Newman , K. Lunnen , G. Wilson , J. Greci , I. Schildkraut , and S. E. V. Phillips , “Crystal Structure of Restriction Endonuclease *Bgl*I Bound to Its Interrupted DNA Recognition Sequence,” EMBO Journal 17, no. 18 (1998): 5466–5476. 9736624 10.1093/emboj/17.18.5466 PMC1170872

[54] M. M. Aboelnga and S. D. Wetmore , “Unveiling a Single‐Metal‐Mediated Phosphodiester Bond Cleavage Mechanism for Nucleic Acids: A Multiscale Computational Investigation of a Human DNA Repair Enzyme,” Journal of the American Chemical Society 141, no. 21 (2019): 8646–8656, 10.1021/jacs.9b03986. 31046259

[55] C. L. Li , L. I. Hor , Z. F. Chang , L. C. Tsai , W. Z. Yang , and H. S. Yuan , “DNA Binding and Cleavage by the Periplasmic Nuclease Vvn: A Novel Structure With a Known Active Site,” EMBO Journal 22, no. 15 (2003): 4014–4025, 10.1093/emboj/cdg377. 12881435 PMC169050

[56] R. Kaur and S. D. Wetmore , “Is Metal Stabilization of the Leaving Group Required or Can Lysine Facilitate Phosphodiester Bond Cleavage in Nucleic Acids? A Computational Study of EndoV,” Journal of Chemical Information and Modeling 64, no. 3 (2024): 944–959, 10.1021/acs.jcim.3c01775. 38253321

[57] N. C. Horton and J. J. Perona , “DNA Cleavage by EcoRV Endonuclease: Two Metal Ions in Three Metal Ion Binding Sites,” Biochemistry 43, no. 22 (2004): 6841–6857, 10.1021/bi0499056. 15170321

[58] Z. Du , J. K. Lee , R. Tjhen , R. M. Stroud , and T. L. James , “Structural and Biochemical Insights Into the Dicing Mechanism of Mouse Dicer: A Conserved Lysine Is Critical for dsRNA Cleavage,” Proceedings of the National Academy of Sciences of the United States of America 105, no. 7 (2008): 2391–2396, 10.1073/pnas.0711506105. 18268334 PMC2268147

[59] H. Zhao , Z. Lin , A. Y. Lynn , et al., “Two Distinct Modes of Metal Ion Binding in the Nuclease Active Site of a Viral DNA‐Packaging Terminase: Insight Into the Two‐Metal‐Ion Catalytic Mechanism,” Nucleic Acids Research 43, no. 22 (2015): 11003–11016. 26450964 10.1093/nar/gkv1018 PMC4678813

[60] T.‐h. Wu , T. Loh , and M. G. Marinus , “The Function of Asp70, Glu77 and Lys79 in the *Escherichia coli* MutH Protein,” Nucleic Acids Research 30, no. 3 (2002): 818–822. 11809896 10.1093/nar/30.3.818 PMC100293

[61] U. Selent , T. Rueter , E. Koehler , et al., “A Site‐Directed Mutagenesis Study to Identify Amino Acid Residues Involved in the Catalytic Function of the Restriction Endonuclease EcoRV,” Biochemistry 31, no. 20 (1992): 4808–4815. 1591242 10.1021/bi00135a010

[62] J. Wu , N. L. Samara , I. Kuraoka , and W. Yang , “Evolution of Inosine‐Specific Endonuclease V From Bacterial DNase to Eukaryotic RNase,” Molecular Cell 76, no. 1 (2019): 44–56, 10.1016/j.molcel.2019.06.046. 31444105 PMC6778043

[63] M. R. Blomberg , T. Borowski , F. Himo , R.‐Z. Liao , and P. E. Siegbahn , “Quantum Chemical Studies of Mechanisms for Metalloenzymes,” Chemical Reviews 114, no. 7 (2014): 3601–3658, 10.1021/cr400388t. 24410477

[64] D. A. Agbaglo , T. J. Summers , Q. Cheng , and N. J. DeYonker , “The Influence of Model Building Schemes and Molecular Dynamics Sampling on QM‐Cluster Models: The Chorismate Mutase Case Study,” Physical Chemistry Chemical Physics 26, no. 16 (2024): 12467–12482, 10.1039/D3CP06100K. 38618904 PMC11090134

[65] X. Sheng and F. Himo , “The Quantum Chemical Cluster Approach in Biocatalysis,” Accounts of Chemical Research 56, no. 8 (2023): 938–947. 36976880 10.1021/acs.accounts.2c00795 PMC10116590

[66] B. Elsässer and P. Goettig , “Mechanisms of Proteolytic Enzymes and Their Inhibition in QM/MM Studies,” International Journal of Molecular Sciences 22, no. 6 (2021): 3232. 33810118 10.3390/ijms22063232 PMC8004986

[67] R. Kaur , D. J. Nikkel , and S. D. Wetmore , “Computational Studies of DNA Repair: Insights Into the Function of Monofunctional DNA Glycosylases in the Base Excision Repair Pathway,” WIREs Computational Molecular Science 10, no. 5 (2020): e1471.

[68] S. F. Sousa , P. A. Fernandes , and M. J. Ramos , “Computational Enzymatic Catalysis – Clarifying Enzymatic Mechanisms With the Help of Computers,” Physical Chemistry Chemical Physics 14, no. 36 (2012): 12431–12441, 10.1039/C2CP41180F. 22870506

[69] R. Gherib , H. M. Dokainish , and J. W. Gauld , “Multi‐Scale Computational Enzymology: Enhancing Our Understanding of Enzymatic Catalysis,” International Journal of Molecular Sciences 15, no. 1 (2014): 401–422, 10.3390/ijms15010401. PMC3907816 24384841

[70] L. Casalino , Ł. Nierzwicki , M. Jinek , and G. Palermo , “Catalytic Mechanism of Non‐Target DNA Cleavage in CRISPR‐Cas9 Revealed by Ab Initio Molecular Dynamics,” ACS Catalysis 10, no. 22 (2020): 13596–13605, 10.1021/acscatal.0c03566. 33520346 PMC7842700

[71] M. E. Alberto , G. Pinto , N. Russo , and M. Toscano , “Triesterase and Promiscuous Diesterase Activities of a Di‐Co^II^‐Containing Organophosphate Degrading Enzyme Reaction Mechanisms,” Chemistry—a European Journal 21, no. 9 (2015): 3736–3745, 10.1002/chem.201405593. 25582757

[72] A. R. Araújo , A. J. M. Ribeiro , P. A. Fernandes , and M. J. Ramos , “Catalytic Mechanism of Retroviral Integrase for the Strand Transfer Reaction Explored by QM/MM Calculations,” Journal of Chemical Theory and Computation 10, no. 12 (2014): 5458–5466, 10.1021/ct500570g. 26583229

[73] M. Rahimian , S. D. Yeole , and S. P. Gejji , “Mechanistic Insights for β‐Cyclodextrin Catalyzed Phosphodiester Hydrolysis,” Journal of Molecular Modeling 20, no. 4 (2014): 2198. 24652502 10.1007/s00894-014-2198-4

[74] H. Xia , W. Zhang , Y. Yang , et al., “Degradation Mechanism of Tris(2‐Chloroethyl) Phosphate (TCEP) as an Emerging Contaminant in Advanced Oxidation Processes: A DFT Modelling Approach,” Chemosphere 273 (2021): 129674, 10.1016/j.chemosphere.2021.129674. 33571912

[75] R. Kaur , A. Frederickson , and S. D. Wetmore , “Elucidation of the Catalytic Mechanism of a Single‐Metal Dependent Homing Endonuclease Using QM and QM/MM Approaches: The Case Study of I‐*Ppo*I,” Physical Chemistry Chemical Physics 26, no. 11 (2024): 8919–8931, 10.1039/D3CP06201E. 38426850

[76] R. Kaur , D. J. Nikkel , M. M. Aboelnga , and S. D. Wetmore , “The Impact of DFT Functional, Cluster Model Size, and Implicit Solvation on the Structural Description of Single‐Metal‐Mediated DNA Phosphodiester Bond Cleavage: The Case Study of APE1,” Journal of Physical Chemistry. B 126, no. 50 (2022): 10672–10683, 10.1021/acs.jpcb.2c06756. 36485014

[77] R. Kaur , M. M. Aboelnga , D. J. Nikkel , and S. D. Wetmore , “The Metal Dependence of Single‐Metal Mediated Phosphodiester Bond Cleavage: A QM/MM Study of a Multifaceted Human Enzyme,” Physical Chemistry Chemical Physics 24, no. 47 (2022): 29130–29140, 10.1039/D2CP04338F. 36444615

[78] B. Elsässer and G. Fels , “Atomistic Details of the Associative Phosphodiester Cleavage in Human Ribonuclease H,” Physical Chemistry Chemical Physics 12, no. 36 (2010): 11081–11088, 10.1039/C001097A. 20672157

[79] S. L. Dürr , O. Bohuszewicz , D. Berta , et al., “The Role of Conserved Residues in the DEDDh Motif: The Proton‐Transfer Mechanism of HIV‐1 RNase H,” ACS Catalysis 11, no. 13 (2021): 7915–7927.

[80] G. Palermo , M. Stenta , A. Cavalli , M. Dal Peraro , and M. De Vivo , “Molecular Simulations Highlight the Role of Metals in Catalysis and Inhibition of Type II Topoisomerase,” Journal of Chemical Theory and Computation 9, no. 2 (2013): 857–862, 10.1021/ct300691u. 26588728

[81] L. Mones , P. Kulhánek , J. Florián , I. Simon , and M. Fuxreiter , “Probing the Two‐Metal Ion Mechanism in the Restriction Endonuclease BamHI,” Biochemistry 46, no. 50 (2007): 14514–14523, 10.1021/bi701630s. 18020376

[82] J. Sgrignani and A. Magistrato , “QM/MM MD Simulations on the Enzymatic Pathway of the Human Flap Endonuclease (hFEN1) Elucidating Common Cleavage Pathways to RNase H Enzymes,” ACS Catalysis 5, no. 6 (2015): 3864–3875, 10.1021/acscatal.5b00178.

[83] D. R. Stevens and S. Hammes‐Schiffer , “Exploring the Role of the Third Active Site Metal Ion in DNA Polymerase η With QM/MM Free Energy Simulations,” Journal of the American Chemical Society 140, no. 28 (2018): 8965–8969, 10.1021/jacs.8b05177. 29932331 PMC6399739

[84] A. J. M. Ribeiro , M. J. Ramos , and P. A. Fernandes , “The Catalytic Mechanism of HIV‐1 Integrase for DNA 3′‐End Processing Established by QM/MM Calculations,” Journal of the American Chemical Society 134, no. 32 (2012): 13436–13447, 10.1021/ja304601k. 22793648

[85] M. Fothergill , M. F. Goodman , J. Petruska , and A. Warshel , “Structure‐Energy Analysis of the Role of Metal Ions in Phosphodiester Bond Hydrolysis by DNA Polymerase I,” Journal of the American Chemical Society 117, no. 47 (1995): 11619–11627, 10.1021/ja00152a001.

[86] S. I. Drusin , R. M. Rasia , and D. M. Moreno , “Study of the Role of Mg^2+^ in dsRNA Processing Mechanism by Bacterial RNase III Through QM/MM Simulations,” Journal of Biological Inorganic Chemistry 25, no. 1 (2020): 89–98, 10.1007/s00775-019-01741-7. 31754801

[87] A. Abyzov , A. Uzun , P. R. Strauss , and V. A. Ilyin , “An AP Endonuclease 1–DNA Polymerase β Complex: Theoretical Prediction of Interacting Surfaces,” PLoS Computational Biology 4, no. 4 (2008): e1000066, 10.1371/journal.pcbi.1000066. 18437203 PMC2289873

[88] O. A. Krumkacheva , G. Y. Shevelev , A. A. Lomzov , et al., “DNA Complexes With Human Apurinic/Apyrimidinic Endonuclease 1: Structural Insights Revealed by Pulsed Dipolar EPR With Orthogonal Spin Labeling,” Nucleic Acids Research 47, no. 15 (2019): 7767–7780. 31329919 10.1093/nar/gkz620 PMC6735896

[89] C. G. P. Doss and N. NagaSundaram , “Investigating the Structural Impacts of I64T and P311S Mutations in APE1‐DNA Complex: A Molecular Dynamics Approach,” PLoS One 7, no. 2 (2012): e31677, 10.1371/journal.pone.0031677. 22384055 PMC3288039

[90] A. B. Guliaev , B. Hang , and B. Singer , “Structural Insights by Molecular Dynamics Simulations Into Specificity of the Major Human AP Endonuclease Toward the Benzene‐Derived DNA Adduct, pBQ‐C,” Nucleic Acids Research 32, no. 9 (2004): 2844–2852, 10.1093/nar/gkh594. 15155853 PMC419600

[91] N. Oezguen , C. H. Schein , S. R. Peddi , T. D. Power , T. Izumi , and W. Braun , “A “Moving Metal Mechanism” for Substrate Cleavage by the DNA Repair Endonuclease APE‐1,” Proteins 68, no. 1 (2007): 313–323, 10.1002/prot.21397. 17427952

[92] N. Oezguen , A. K. Mantha , T. Izumi , C. H. Schein , S. Mitra , and W. Braun , “MD Simulation and Experimental Evidence for Mg^2+^ Binding at the B Site in Human AP Endonuclease 1,” Bioinformation 7, no. 4 (2011): 184–198, 10.6026/97320630007184. 22102776 PMC3218521

[93] H. Batebi , J. Dragelj , and P. Imhof , “Role of AP‐Endonuclease (Ape1) Active Site Residues in Stabilization of the Reactant Enzyme‐DNA Complex,” Proteins 86, no. 4 (2018): 439–453. 29344998 10.1002/prot.25460

[94] L. W. Chung , W. M. C. Sameera , R. Ramozzi , et al., “The ONIOM Method and Its Applications,” Chemical Reviews 115, no. 12 (2015): 5678–5796, 10.1021/cr5004419. 25853797

[95] R. L. Setten , J. J. Rossi , and S.‐p. Han , “The Current State and Future Directions of RNAi‐Based Therapeutics,” Nature Reviews. Drug Discovery 18, no. 6 (2019): 421–446, 10.1038/s41573-019-0017-4. 30846871

[96] Y. Weng , H. Xiao , J. Zhang , X.‐J. Liang , and Y. Huang , “RNAi Therapeutic and Its Innovative Biotechnological Evolution,” Biotechnology Advances 37, no. 5 (2019): 801–825, 10.1016/j.biotechadv.2019.04.012. 31034960

[97] W. M. Merritt , M. Bar‐Eli , and A. K. Sood , “The Dicey Role of Dicer: Implications for RNAi Therapy,” Cancer Research 70, no. 7 (2010): 2571–2574. 20179193 10.1158/0008-5472.CAN-09-2536 PMC3170915

[98] J. M. Casacuberta , Y. Devos , P. du Jardin , M. Ramon , H. Vaucheret , and F. Nogué , “Biotechnological Uses of RNAi in Plants: Risk Assessment Considerations,” Trends in Biotechnology 33, no. 3 (2015): 145–147, 10.1016/j.tibtech.2014.12.003. 25721261

[99] C. E. Moreira‐Pinto , R. R. Coelho , A. G. B. Leite , et al., “Increasing *Anthonomus grandis* Susceptibility to Metarhizium Anisopliae Through RNAi‐Induced AgraRelish Knockdown: A Perspective to Combine Biocontrol and Biotechnology,” Pest Management Science 77, no. 9 (2021): 4054–4063. 33896113 10.1002/ps.6430

[100] J. H. Sherman , T. Munyikwa , S. Y. Chan , J. S. Petrick , K. W. Witwer , and S. Choudhuri , “RNAi Technologies in Agricultural Biotechnology: The Toxicology Forum 40th Annual Summer Meeting,” Regulatory Toxicology and Pharmacology 73, no. 2 (2015): 671–680. 26361858 10.1016/j.yrtph.2015.09.001

[101] Consortium, T. U , “UniProt: The Universal Protein Knowledgebase in 2025,” Nucleic Acids Research 53, no. D1 (2024): D609–D617. 10.1093/nar/gkae1010 PMC11701636 39552041

[102] Schrodinger, LLC , “The PyMOL Molecular Graphics System, Version 1.8,” (2015).

[103] A. Waterhouse , M. Bertoni , S. Bienert , et al., “SWISS‐MODEL: Homology Modelling of Protein Structures and Complexes,” Nucleic Acids Research 46 (2018): W296–W303. 29788355 10.1093/nar/gky427 PMC6030848

[104] R. Anandakrishnan , B. Aguilar , and A. V. Onufriev , “H++ 3.0: Automating pK Prediction and the Preparation of Biomolecular Structures for Atomistic Molecular Modeling and Simulations,” Nucleic Acids Research 40, no. W1 (2012): W537–W541. 22570416 10.1093/nar/gks375 PMC3394296

[105] J. C. Gordon , J. B. Myers , T. Folta , V. Shoja , L. S. Heath , and A. Onufriev , “H++: A Server for Estimating p Ka s and Adding Missing Hydrogens to Macromolecules,” Nucleic Acids Research 33 (2005): W368–W371. 15980491 10.1093/nar/gki464 PMC1160225

[106] D. A. Case , I. Y. Ben‐Shalom , S. R. Brozell , et al., “AMBER 2018, University of California,” 2018.

[107] J. A. Maier , C. Martinez , K. Kasavajhala , L. Wickstrom , K. E. Hauser , and C. Simmerling , “ff14SB: Improving the Accuracy of Protein Side Chain and Backbone Parameters From ff99SB,” Journal of Chemical Theory and Computation 11, no. 8 (2015): 3696–3713, 10.1021/acs.jctc.5b00255. 26574453 PMC4821407

[108] A. Pérez , I. Marchán , D. Svozil , et al., “Refinement of the AMBER Force Field for Nucleic Acids: Improving the Description of α/γ Conformers,” Biophysical Journal 92, no. 11 (2007): 3817–3829. 17351000 10.1529/biophysj.106.097782 PMC1868997

[109] M. Zgarbová , M. Otyepka , J. Šponer , et al., “Refinement of the Cornell Et al. Nucleic Acids Force Field Based on Reference Quantum Chemical Calculations of Glycosidic Torsion Profiles,” Journal of Chemical Theory and Computation 7, no. 9 (2011): 2886–2902, 10.1021/ct200162x. 21921995 PMC3171997

[110] F.‐Y. Dupradeau , A. Pigache , T. Zaffran , et al., “The R.E.D. Tools: Advances in RESP and ESP Charge Derivation and Force Field Library Building,” Physical Chemistry Chemical Physics 12, no. 28 (2010): 7821–7839, 10.1039/c0cp00111b. 20574571 PMC2918240

[111] T. Vreven and K. Morokuma , “The ONIOM (Our Own N‐Layered Integrated Molecular Orbital + Molecular Mechanics) Method for the First Singlet Excited (S1) State Photoisomerization Path of a Retinal Protonated Schiff Base,” Journal of Chemical Physics 113, no. 8 (2000): 2969–2975, 10.1063/1.1287059.

[112] Y. Zhao and D. G. Truhlar , “The M06 Suite of Density Functionals for Main Group Thermochemistry, Thermochemical Kinetics, Noncovalent Interactions, Excited States, and Transition Elements: Two New Functionals and Systematic Testing of Four M06‐Class Functionals and 12 Other Functionals,” Theoretical Chemistry Accounts 120, no. 1 (2008): 215–241, 10.1007/s00214-007-0310-x.

[113] S. Pilbák , Ö. Farkas , and L. Poppe , “Mechanism of the Tyrosine Ammonia Lyase Reaction—Tandem Nucleophilic and Electrophilic Enhancement by a Proton Transfer,” Chemistry ‐ A European Journal 18, no. 25 (2012): 7793–7802, 10.1002/chem.201103662. 22573540

[114] X. Hu , H. Hu , J. A. Melvin , K. W. Clancy , D. G. McCafferty , and W. Yang , “Autocatalytic Intramolecular Isopeptide Bond Formation in Gram‐Positive Bacterial Pili: A QM/MM Simulation,” Journal of the American Chemical Society 133, no. 3 (2011): 478–485, 10.1021/ja107513t. 21142157 PMC3081525

[115] S. Wu , D. Xu , and H. Guo , “QM/MM Studies of Monozinc β‐Lactamase CphA Suggest That the Crystal Structure of an Enzyme−Intermediate Complex Represents a Minor Pathway,” Journal of the American Chemical Society 132, no. 51 (2010): 17986–17988, 10.1021/ja104241g. 21138257 PMC3009838

[116] D. J. Nikkel and S. D. Wetmore , “Distinctive Formation of a DNA–Protein Cross‐Link During the Repair of DNA Oxidative Damage: Insights Into Human Disease From MD Simulations and QM/MM Calculations,” Journal of the American Chemical Society 145, no. 24 (2023): 13114–13125, 10.1021/jacs.3c01773. 37285289

[117] E. Ma , I. J. MacRae , J. F. Kirsch , and J. A. Doudna , “Autoinhibition of Human Dicer by Its Internal Helicase Domain,” Journal of Molecular Biology 380, no. 1 (2008): 237–243, 10.1016/j.jmb.2008.05.005. 18508075 PMC2927216

[118] S. Chakravarthy , S. H. Sternberg , C. A. Kellenberger , and J. A. Doudna , “Substrate‐Specific Kinetics of Dicer‐Catalyzed RNA Processing,” Journal of Molecular Biology 404, no. 3 (2010): 392–402. 20932845 10.1016/j.jmb.2010.09.030 PMC3005596

[119] J. Bouvette , D. N. Korkut , A. Fouillen , et al., “High‐Yield Production of Human Dicer by Transfection of Human HEK293‐EBNA1 Cells Grown in Suspension,” BMC Biotechnology 18, no. 1 (2018): 76, 10.1186/s12896-018-0485-3. 30522464 PMC6282390

[120] M. J. Frisch , G. W. Trucks , H. B. Schlegel , et al., Gaussian 16 Rev. B.01 (Gaussian, Inc., 2016).

[121] C. Jackson , H.‐K. Kim , P. D. Carr , J.‐W. Liu , and D. L. Ollis , “The Structure of an Enzyme–Product Complex Reveals the Critical Role of a Terminal Hydroxide Nucleophile in the Bacterial Phosphotriesterase Mechanism,” Biochimica et Biophysica Acta (BBA)‐Proteins and Proteomics 1752, no. 1 (2005): 56–64, 10.1016/j.bbapap.2005.06.008. 16054447

[122] E. R. Morris , S. J. Caswell , S. Kunzelmann , et al., “Crystal Structures of SAMHD1 Inhibitor Complexes Reveal the Mechanism of Water‐Mediated dNTP Hydrolysis,” Nature Communications 11, no. 1 (2020): 3165, 10.1038/s41467-020-16983-2. PMC7311409 32576829

[123] M.‐K. Hong , A. J. M. Ribeiro , J.‐K. Kim , et al., “Divalent Metal Ion‐Based Catalytic Mechanism of the Nudix Hydrolase Orf153 (YmfB) From *Escherichia coli* ,” Acta Crystallographica, Section D: Biological Crystallography 70, no. 5 (2014): 1297–1310. 24816099 10.1107/S1399004714002570

[124] A. T. P. Carvalho , P. A. Fernandes , and M. J. Ramos , “The Catalytic Mechanism of RNA Polymerase II,” Journal of Chemical Theory and Computation 7, no. 4 (2011): 1177–1188, 10.1021/ct100579w. 26606364

[125] H. He , Q. Chen , and M. M. Georgiadis , “High‐Resolution Crystal Structures Reveal Plasticity in the Metal Binding Site of Apurinic/Apyrimidinic Endonuclease I,” Biochemistry 53, no. 41 (2014): 6520–6529, 10.1021/bi500676p. 25251148 PMC4204877

[126] A. Gutteridge and J. Thornton , “Conformational Changes Observed in Enzyme Crystal Structures Upon Substrate Binding,” Journal of Molecular Biology 346, no. 1 (2005): 21–28, 10.1016/j.jmb.2004.11.013. 15663924

[127] M. Newman , J. Murray‐Rust , J. Lally , et al., “Structure of an XPF Endonuclease With and Without DNA Suggests a Model for Substrate Recognition,” EMBO Journal 24, no. 5 (2005): 895–905, 10.1038/sj.emboj.7600581. 15719018 PMC554130

[128] C.‐Y. Kim , M. S. Park , and R. B. Dyer , “Human Flap Endonuclease‐1: Conformational Change Upon Binding to the Flap DNA Substrate and Location of the Mg^2+^ Binding Site,” Biochemistry 40, no. 10 (2001): 3208–3214, 10.1021/bi002100n. 11258937

[129] B. Zhou , L. Zheng , B. Wu , et al., “A Conditional Protein Diffusion Model Generates Artificial Programmable Endonuclease Sequences With Enhanced Activity,” Cell Discovery 10, no. 1 (2024): 95, 10.1038/s41421-024-00728-2. 39251570 PMC11385924

[130] J. Smith , S. Grizot , S. Arnould , et al., “A Combinatorial Approach to Create Artificial Homing Endonucleases Cleaving Chosen Sequences,” Nucleic Acids Research 34, no. 22 (2006): e149. 17130168 10.1093/nar/gkl720 PMC1702487

[131] H. Yone , H. Kono , M. Sato , and K. Ohta , “AlphaFold3‐Guided Optimization of a Photoactivatable Endonuclease for Top‐Down Genome Engineering,” Journal of Biological Chemistry 301, no. 11 (2025): 110762, 10.1016/j.jbc.2025.110762. 41005475 PMC12569821

[132] D. W. Binzel , S. Guo , H. Yin , et al., “Rational Design for Controlled Release of Dicer‐Substrate siRNA Harbored in phi29 pRNA‐Based Nanoparticles,” Molecular Therapy—Nucleic Acids 25 (2021): 524–535, 10.1016/j.omtn.2021.07.021. 34589275 PMC8463318

[133] M. A. G. Raja , H. Katas , and M. W. Amjad , “Design, Mechanism, Delivery and Therapeutics of Canonical and Dicer‐Substrate siRNA,” Asian Journal of Pharmaceutical Sciences 14, no. 5 (2019): 497–510, 10.1016/j.ajps.2018.12.005. 32104477 PMC7032099

[134] D. Rakheja , K. S. Chen , Y. Liu , et al., “Somatic Mutations in DROSHA and DICER1 Impair microRNA Biogenesis Through Distinct Mechanisms in Wilms Tumours,” Nature Communications 5, no. 1 (2014): 4802, 10.1038/ncomms5802. PMC4159681 25190313

[135] L. Fernández‐Martínez , J. A. Villegas , Í. Santamaría , et al., “Identification of Somatic and Germ‐Line DICER1 Mutations in Pleuropulmonary Blastoma, Cystic Nephroma and Rhabdomyosarcoma Tumors Within a DICER1 Syndrome Pedigree,” BMC Cancer 17, no. 1 (2017): 146, 10.1186/s12885-017-3136-5. 28222777 PMC5320664

[136] M. Apellaniz‐Ruiz , M. Segni , M. Kettwig , et al., “Mesenchymal Hamartoma of the Liver and DICER1 Syndrome,” New England Journal of Medicine 380, no. 19 (2019): 1834–1842, 10.1056/nejmoa1812169. 31067372

