# Supplementary material for: How Do DICER1 Syndrome Mutations Disrupt Catalysis? Unveiling Dicer Metal Binding Architecture and Mechanism of Action Using MD Simulations and QM/MM Calculations
Source: J Comput Chem. 2026 Jul 3;47(18):e70446. doi: 10.1002/jcc.70446 (PMC13334501; doi:10.1002/jcc.70446)
Supplement: Supplementary file 1 — Figure S1: Dicer structure, highlighting the various domains. Figure S2: Overlay of mouse (purple, PDB ID: 3C4B) and human (green, PDB ID: 2EB1) Dicer RNase IIIb domains highlighting (A) the overall structural similarity and (B) the similar positioning of K1806 and K1790. Crystal structures of (C) the K38A mutant EcoRV bound to DNA, (D) MutH bound to DNA, (E) Endo V, and (F) the gp2 subunit of Sf6, showing a similarly positioned active site lysine as in Dicer (K1806). Figure S3: Sequence alignment of bacterial RNase III and the Dicer RNase IIIb domain. Mg2+‐binding regions underlined in red. Figure S4: (A) Overlay of aa‐RNase III (magenta) and Dicer RNase IIIb homodimer (PDB ID: 2 EB1, green), highlighting key active site residues and differing locations of Mg2+. Overlays of the cryo‐EM structure of Ca2+−inhibited Dicer (cyan, PDB ID: 7XW2) and (B) the post‐equilibration aaRIII model (magenta), and (C) the post‐equilibration RIIIb model (green). Figure S5: All‐heavy‐atom RMSD relative to the first frame over the MD simulations for the (A) aaRIII and (B) RIIIb wild‐type Dicer models. Five 500 ns replicates are joined back‐to‐back. Figure S6: All‐heavy‐atom RMSD relative to the first frame over the MD simulations for the (A) D1709N, (B) D1810Y, (C) E1813D, (D) E1705K, (E) E1813G, and (F) G1809R Dicer mutants. Five 500 ns replicates are joined back‐to‐back. Figure S7: QM/MM RIIIb (left) and aaRIII (right) models used to calculate the Dicer catalytic pathway. QM region highlighted in blue. Figure S8: (A) Overlay of key ONIOM(M06‐2X/6‐311+G(2df,p):AMBERff14SB)//ONIOM(M06‐2X/6‐31G(d,p):AMBERff14SB) stationary points for the Dicer catalytic mechanism obtained using the aaRIII model employing the mechanical (magenta) and electronic (cyan) embedding schemes during geometry optimization. Calculated Dicer mechanism using (B) electronic embedding and (C) mechanical embedding during optimizations for the aaRIII model. Distances and RMSDs are reported in Å. Relative energies are [file JCC-47-0-s001.docx]

**How Do DICER1 Syndrome Mutations Disrupt Catalysis? Unveiling Dicer Metal Binding Architecture and Mechanism of Action using MD Simulations and QM/MM Calculations**

Dylan J. Nikkel and Stacey D. Wetmore*

Department of Chemistry and Biochemistry, University of Lethbridge, Lethbridge, Alberta, Canada T1K 3M4

Supporting Information

(16 Pages)

**Contents**

**Figure** **S1.** Dicer structure, highlighting the various domains S3

**Figure S2.** Overlay of mouse (purple, PDB ID: 3C4B) and human (green, PDB ID: 2EB1) Dicer RNase IIIb domains highlighting A) the overall structural similarity and B) the similar positioning of K1806 and K1790. Crystal structures of C) the K38A mutant EcoRV bound to DNA, D) MutH bound to DNA, E) Endo V, and F) the gp2 subunit of Sf6, showing a similarly positioned active site lysine as in Dicer (K1806) S4

**Figure S3.** Sequence alignment of bacterial RNase III and the Dicer RNase IIIb domain. Mg^2+^-binding regions underlined in red.…………...………………………………………………………………………………………………………S5

**Figure S4.** A) Overlay of *aa*-RNase III (magenta) and Dicer RNase IIIb homodimer (PDB ID: 2EB1, green), highlighting key active site residues and differing locations of Mg^2+^. Overlays of the cryo-EM structure of Ca^2+^−inhibited Dicer (cyan, PDB ID: 7XW2) and B) the post-equilibration aaRIII model (magenta), and C) the post-equilibration RIIIb model (green). S5

**Figure S5.** All-heavy-atom RMSD relative to the first frame over the MD simulations for the A) aaRIII and B) RIIIb wild-type Dicer models. Five 500 ns replicates are joined back-to-back. S6

**Figure S6.** All-heavy-atom RMSD relative to the first frame over the MD simulations for the A) D1709N, B) D1810Y, C) E1813D, D) E1705K, E) E1813G, and F) G1809R Dicer mutants. Five 500 ns replicates are joined back-to-back S6

**Figure S7.** QM/MM RIIIb (left) and aaRIII (right) models used to calculate the Dicer catalytic pathway. QM region highlighted in blue S7

**Figure S8.** A) Overlay of key ONIOM(M06-2X/6-311+G(2df,p):AMBERff14SB)//ONIOM(M06-2X/6-31G(d,p):AMBERff14SB) stationary points for the Dicer catalytic mechanism obtained using the aaRIII model employing the mechanical (magenta) and electronic (cyan) embedding schemes during geometry optimization. Calculated Dicer mechanism using B) electronic embedding and C) mechanical embedding during optimizations for the aaRIII model. Distances and RMSDs are reported in Å. Relative energies are reported in kJ/mol S8

**Figure S9.** Histogram of the fractional occupancy of the distance (Å) between Mg_B_^2+^ and the substrate O3′ leaving group, and the ∠(OδCγCβCα) dihedral angle of D1709 (°) over the MD simulations for the RIIIb model. MD representative structures of conformations can be found in Figure 5 S9

**Figure S10.** ONIOM(M06-2X/6-31G(d,p):AMBERff14SB) optimized reactant complexes, highlighting Mg_A_^2+^ (left) and Mg_B_^2+^ (right) coordination for the A) RIIIb and B) aaRIII models involving a water nucleophile, and C) aaRIII model involving a hydroxide nucleophile S10

**Figure S11.** ONIOM(M06-2X/6-311+G(2df,p):AMBERff14SB)//ONIOM(M06-2X/6-31G(d,p):AMBERff14SB) calculated Dicer catalytic mechanism involving a water nucleophile obtained using the RIIIb model. Distances reported in Å. Water molecules coordinated to Mg^2+^ ions that do not participate in the reaction are omitted for clarity S11

**Figure S12.** Histogram of the fractional occupancies of the distance (Å) between Mg_A_^2+^ or Mg_B_^2+^ and the substrate non-bridging phosphate oxygen over the MD simulations for the aaRIII model. MD representative structures of conformations can be found in Figure 7 S12

**Figure S13.** Average ∂V/∂λ from thermodynamic integration at different λ values for the alchemical transformation of water into hydroxide in A) Dicer and B) bulk solvent S13

**Figure S14.** ONIOM(M06-2X/6-311+G(2df,p):AMBERff14SB)//ONIOM(M06-2X/6-31G(d,p):AMBERff14SB) calculated Dicer catalytic mechanism involving a hydroxide nucleophile obtained using the aaRIII model. Distances reported in Å S13

**Figure S15.** Overlay of MD representative structures of the active (2Mg^2+^−B) conformation of the Dicer aaRIII model containing a water (magenta) and hydroxide (white) nucleophile coordinated to Mg_A_^2+^. RMSD is reported in Å S14

**Figure S16.** MD representative structures for the 2Mg^2+^−B (left), Mg_A_^2+^−U (middle), and Mg_B_^2+^−U (right) conformations for the A) D1709N, B) G1809R, C) E1813G, D) E1705K, E) D1810Y, and F) E1813D Dicer mutants S15

**Table S1.** RESP charges for a hydroxide ion calculated at the HF/6-31G(d) level of theory S16

**Table S2.** Relative energies of key stationary points for the RIIIb and aaRIII Dicer models S16

**Scheme S1.** Thermodynamic cycle used to calculate the ΔΔG_Binding_ for replacing water with hydroxide in the Dicer active site………………………………………………………………………………………………………S16

**Equation S1**…………………………..…………………………………..…………………………………………………………………S16


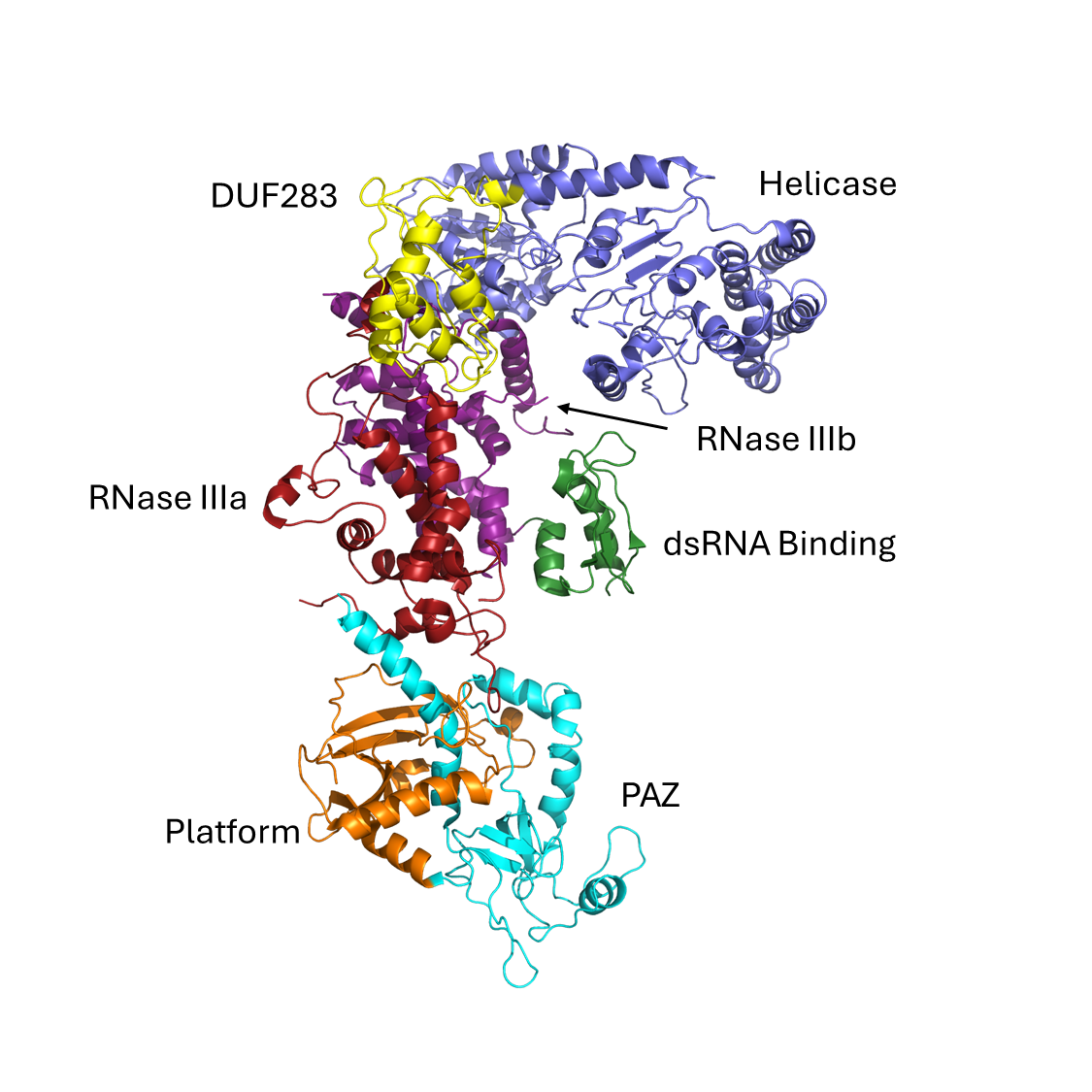


**Figure** **S1.** Dicer structure, highlighting the various domains.


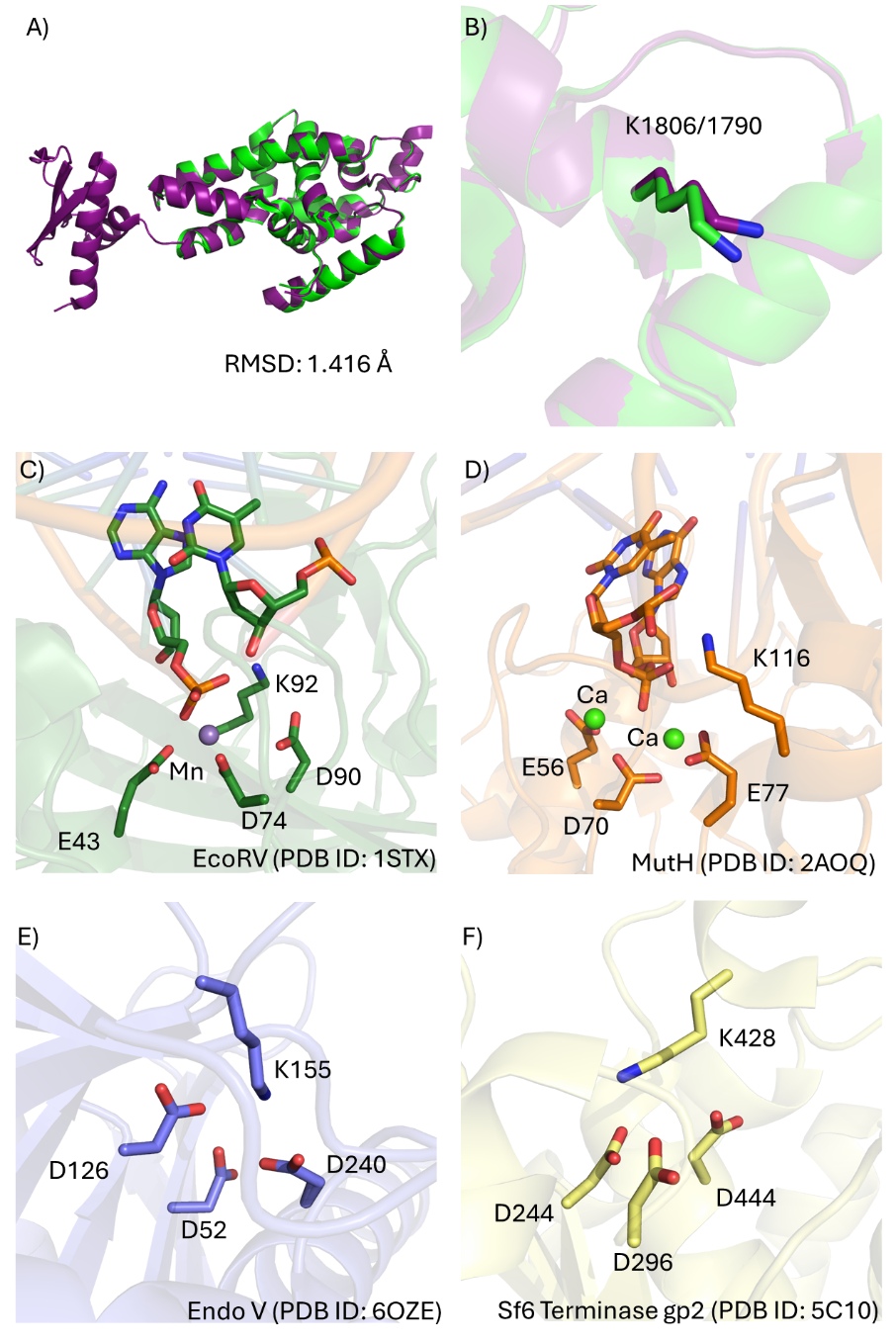


**Figure S2.** Overlay of mouse (purple, PDB ID: 3C4B) and human (green, PDB ID: 2EB1) Dicer RNase IIIb domains highlighting A) the overall structural similarity and B) the similar positioning of K1806 and K1790. Crystal structures of C) the K38A mutant EcoRV bound to DNA, D) MutH bound to DNA, E) human Endo V, and F) the gp2 subunit of Sf6, showing a similarly positioned active site lysine as in Dicer (K1806).


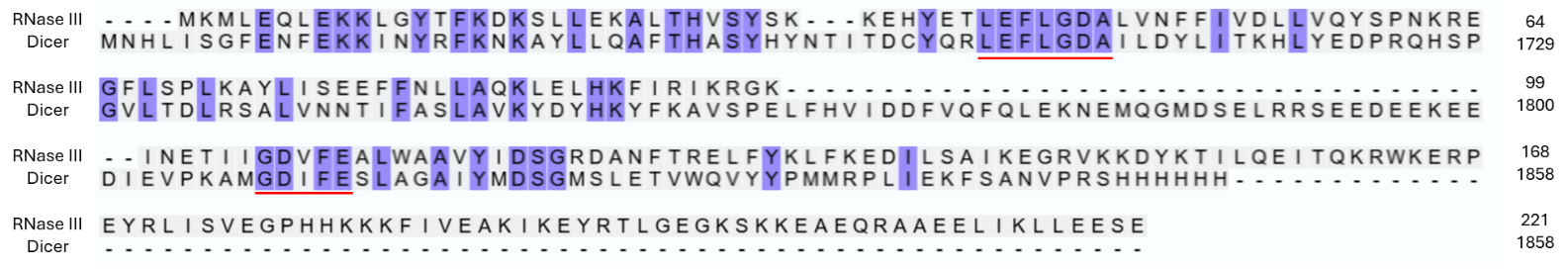


**Figure S3.** Sequence alignment of bacterial RNase III and the Dicer RNase IIIb domain. Mg^2+^-binding regions underlined in red.


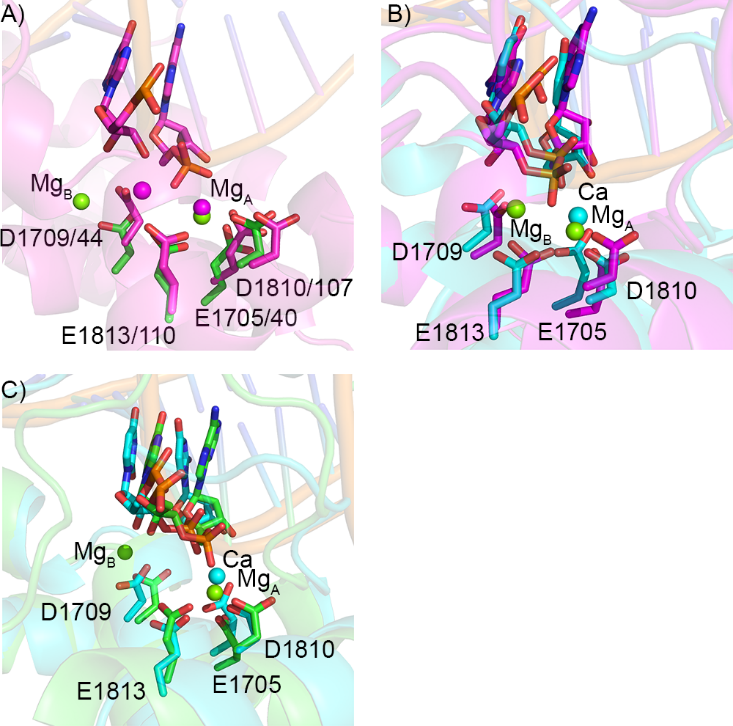


**Figure S4.** A) Overlay of *aa*-RNase III (magenta) and Dicer RNase IIIb homodimer (PDB ID: 2EB1, green), highlighting key active site residues and differing locations of Mg^2+^. Overlays of the cryo-EM structure of Ca^2+^−inhibited Dicer (cyan, PDB ID: 7XW2) and B) the post-equilibration aaRIII model (magenta), and C) the post-equilibration RIIIb model (green).


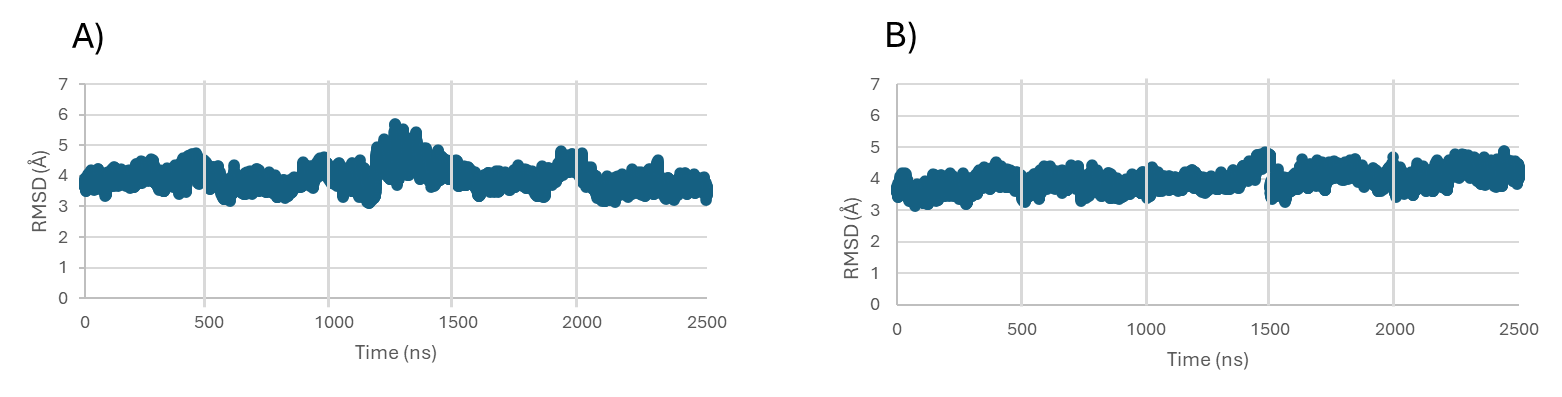


**Figure S5.** All-heavy-atom RMSD relative to the first frame over the MD simulations for the A) aaRIII and B) RIIIb wild-type Dicer models. Five 500 ns replicates are joined back-to-back. The RMSD was calculated with respect to the first frame of the 100 ns MD simulation equilibration step.


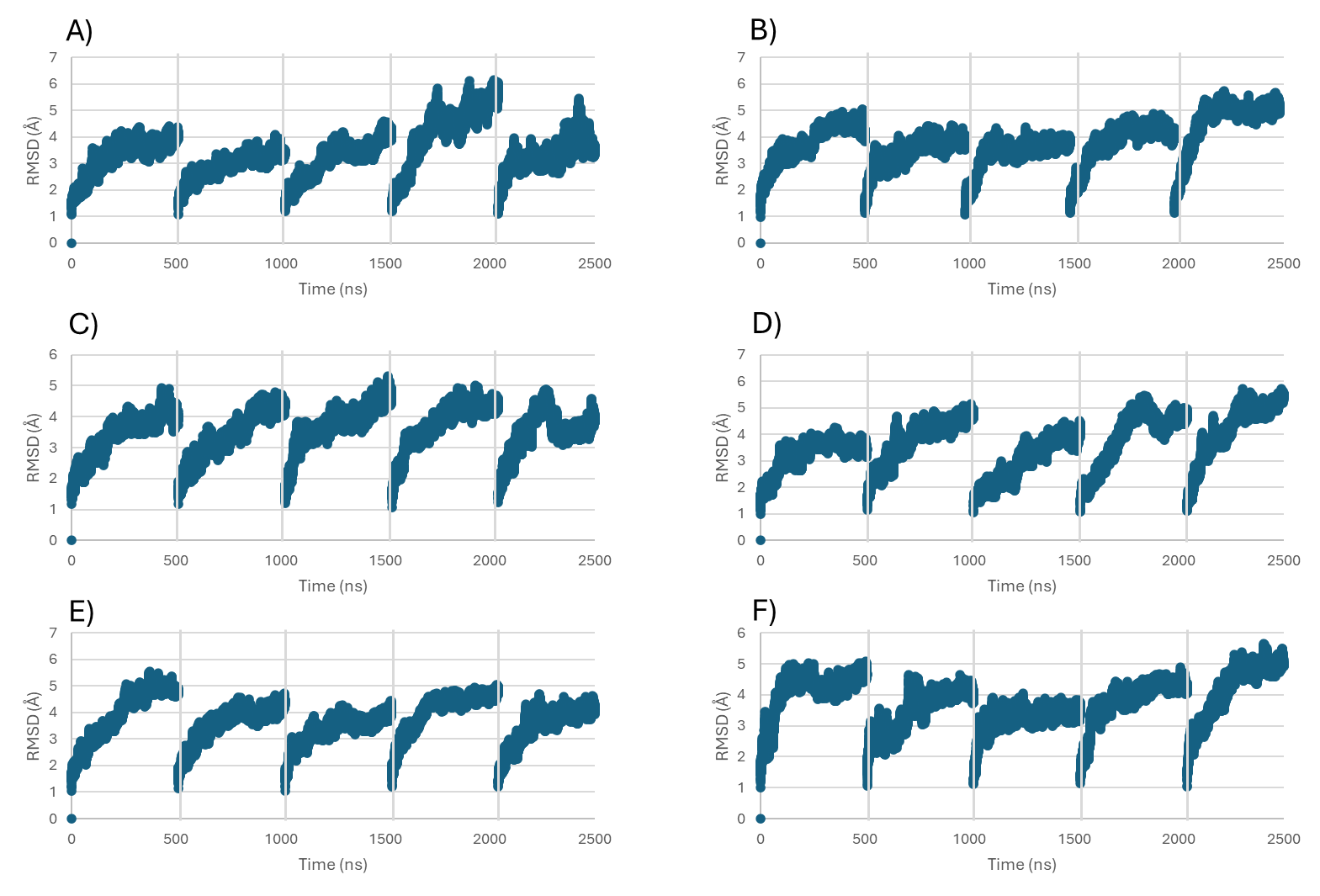


**Figure S6.** All-heavy-atom RMSD relative to the first frame over the MD simulations for the A) D1709N, B) D1810Y, C) E1813D, D) E1705K, E) E1813G, and F) G1809R Dicer mutants. Five 500 ns replicates are joined back-to-back. The RMSD was calculated with respect to the first frame of the production simulations.


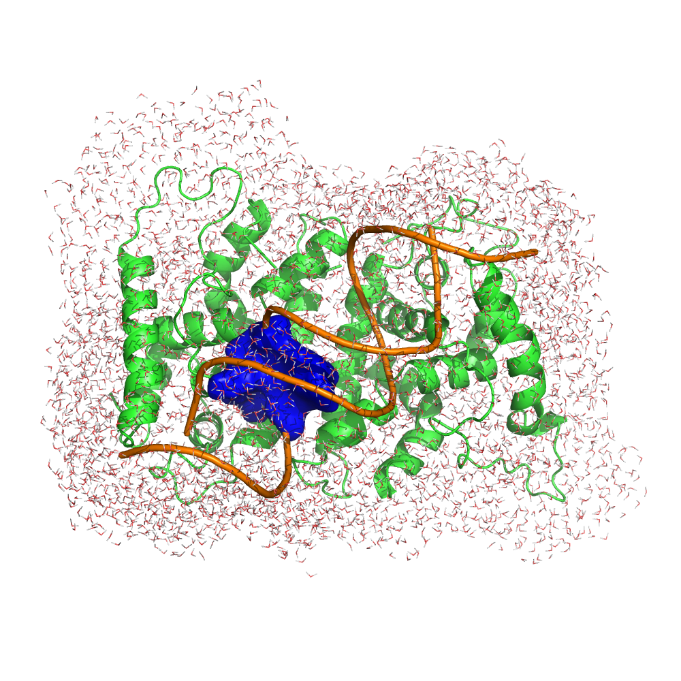

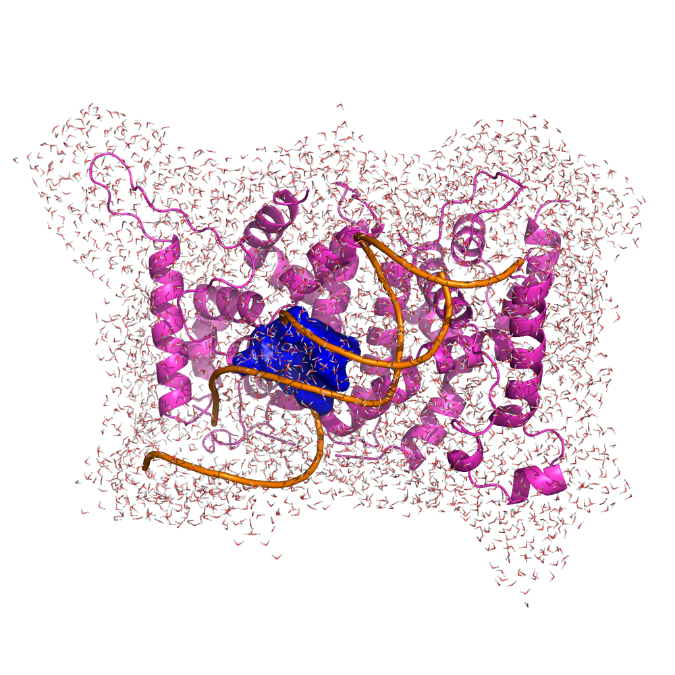


**Figure S7.** QM/MM RIIIb (left) and aaRIII (right) models used to calculate the Dicer catalytic pathway. QM region highlighted in blue.


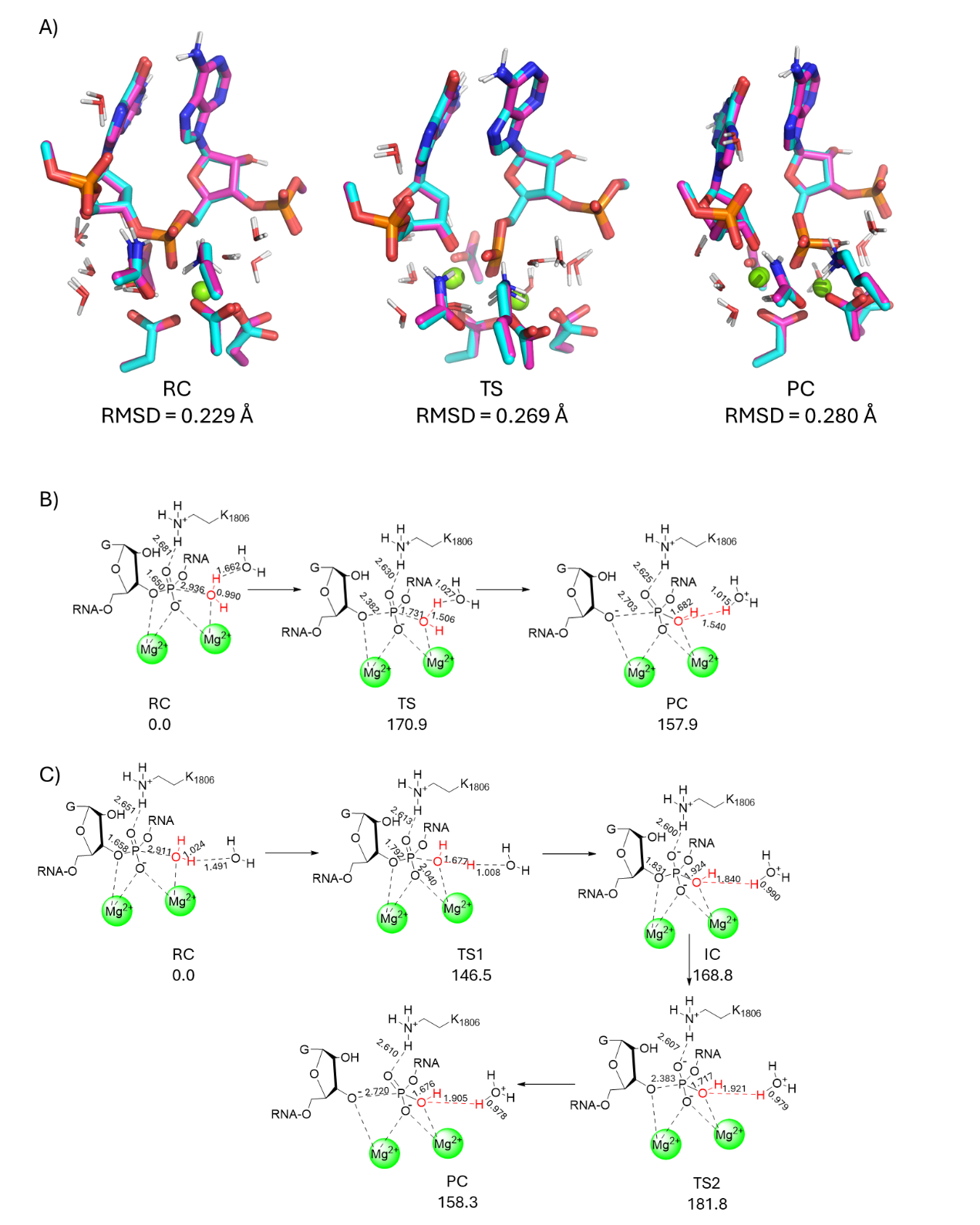


**Figure S8.** A) Overlay of key ONIOM(M06-2X/6-311+G(2df,p):AMBERff14SB)//ONIOM(M06-2X/6-31G(d,p):AMBERff14SB) stationary points for the Dicer catalytic mechanism obtained using the aaRIII model employing the mechanical (magenta) and electronic (cyan) embedding schemes during geometry optimization. Calculated Dicer mechanism using B) electronic embedding and C) mechanical embedding during optimizations for the aaRIII model. Distances and RMSDs are reported in Å. Relative energies are reported in kJ/mol.


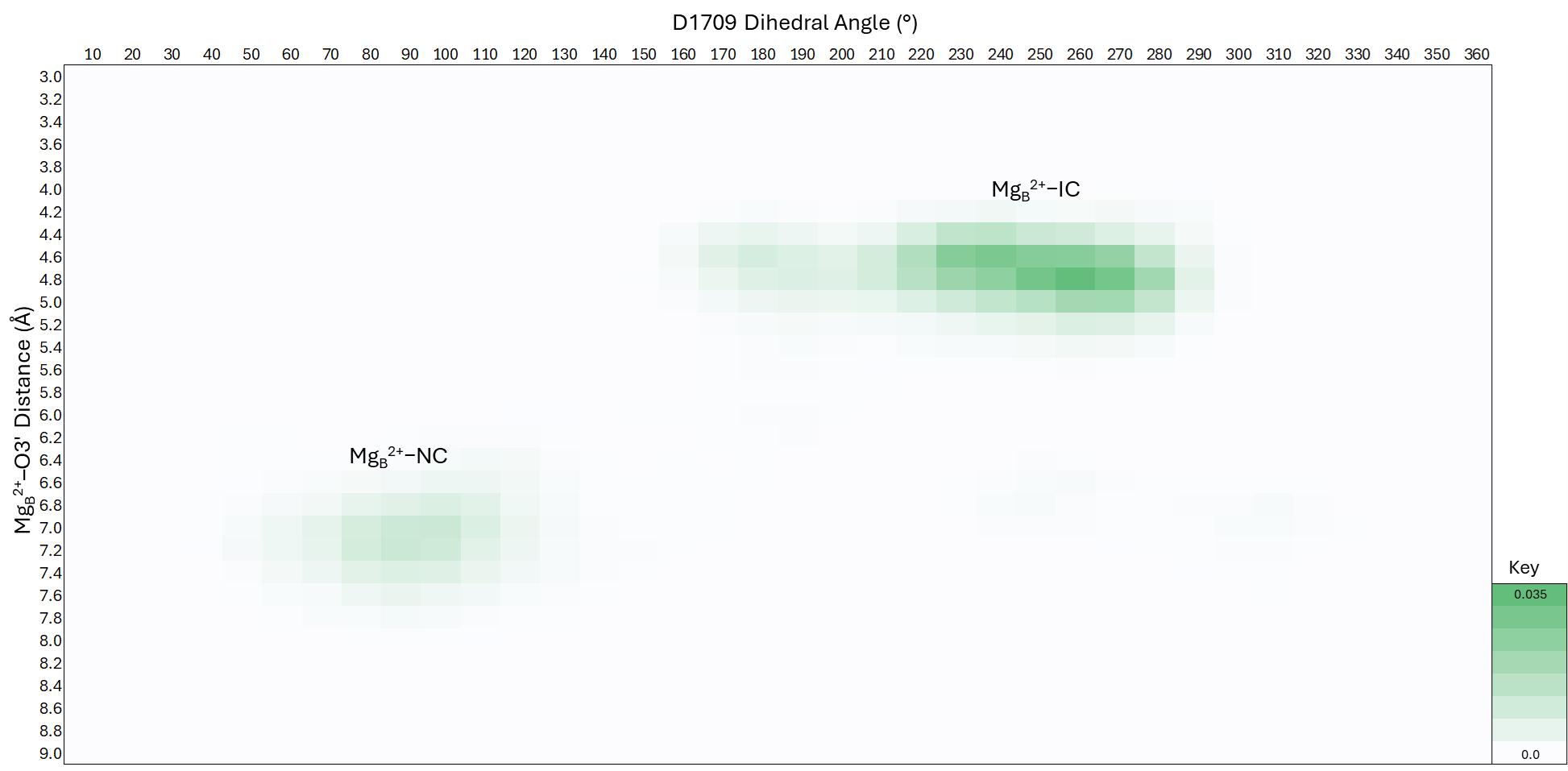


**Figure S9.** Histogram of the fractional occupancy of the distance (Å) between Mg_B_^2+^ and the substrate O3′ leaving group, and the ∠(OδCγCβCα) dihedral angle of D1709 (°) over the MD simulations for the RIIIb model. MD representative structures of conformations can be found in Figure 5.


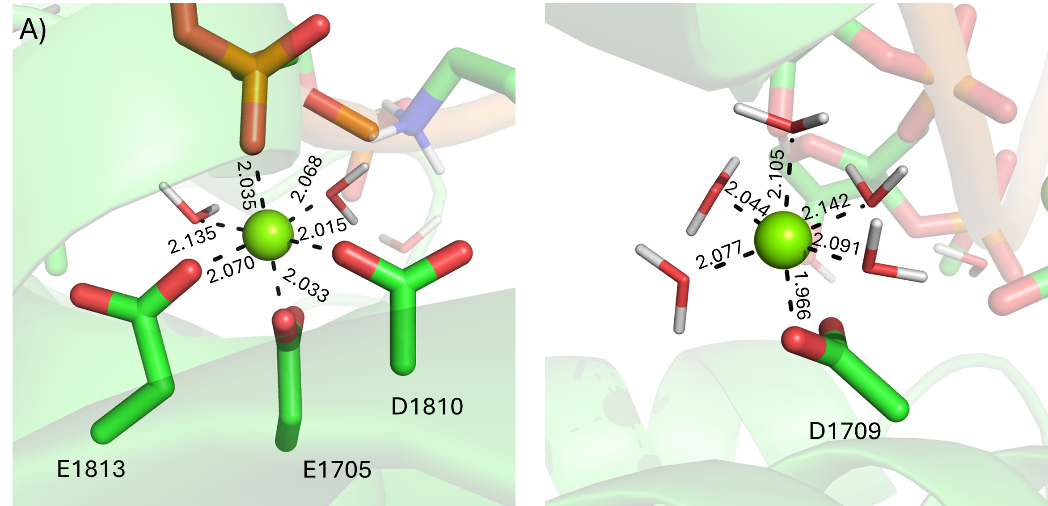


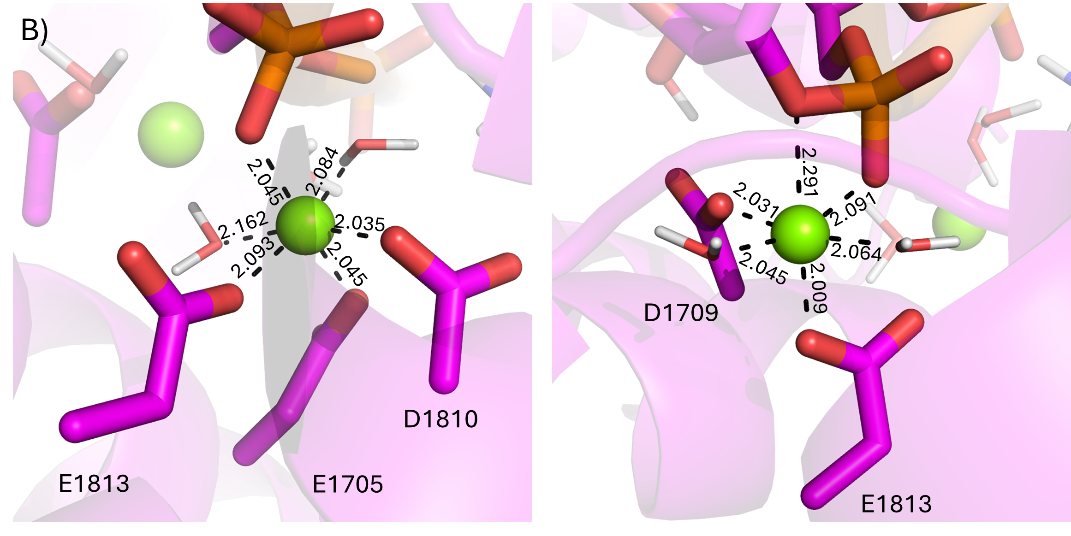


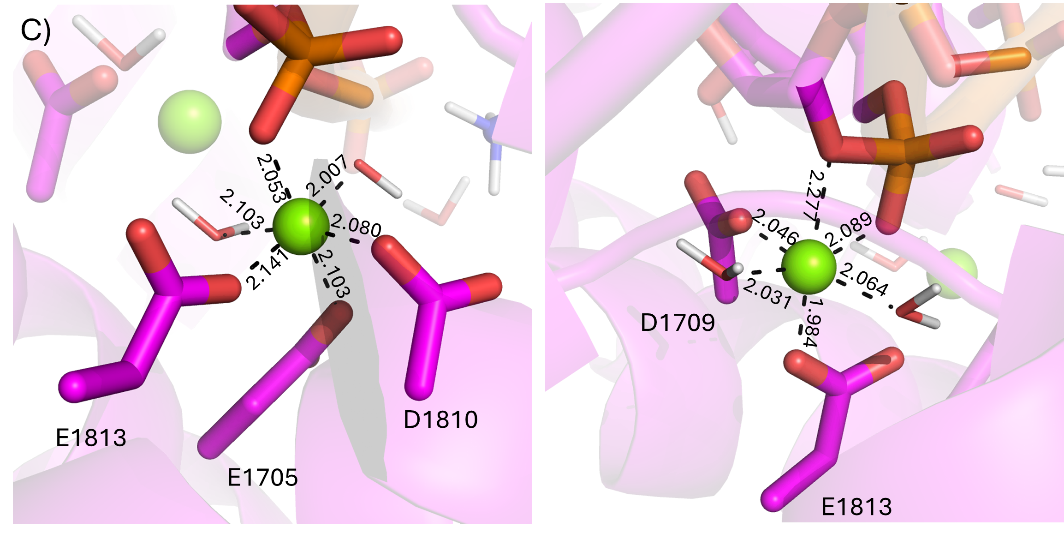


**Figure S10.** ONIOM(M06-2X/6-31G(d,p):AMBERff14SB) optimized reactant complexes, highlighting Mg_A_^2+^ (left) and Mg_B_^2+^ (right) coordination for the A) RIIIb and B) aaRIII models involving a water nucleophile, and C) aaRIII model involving a hydroxide nucleophile.


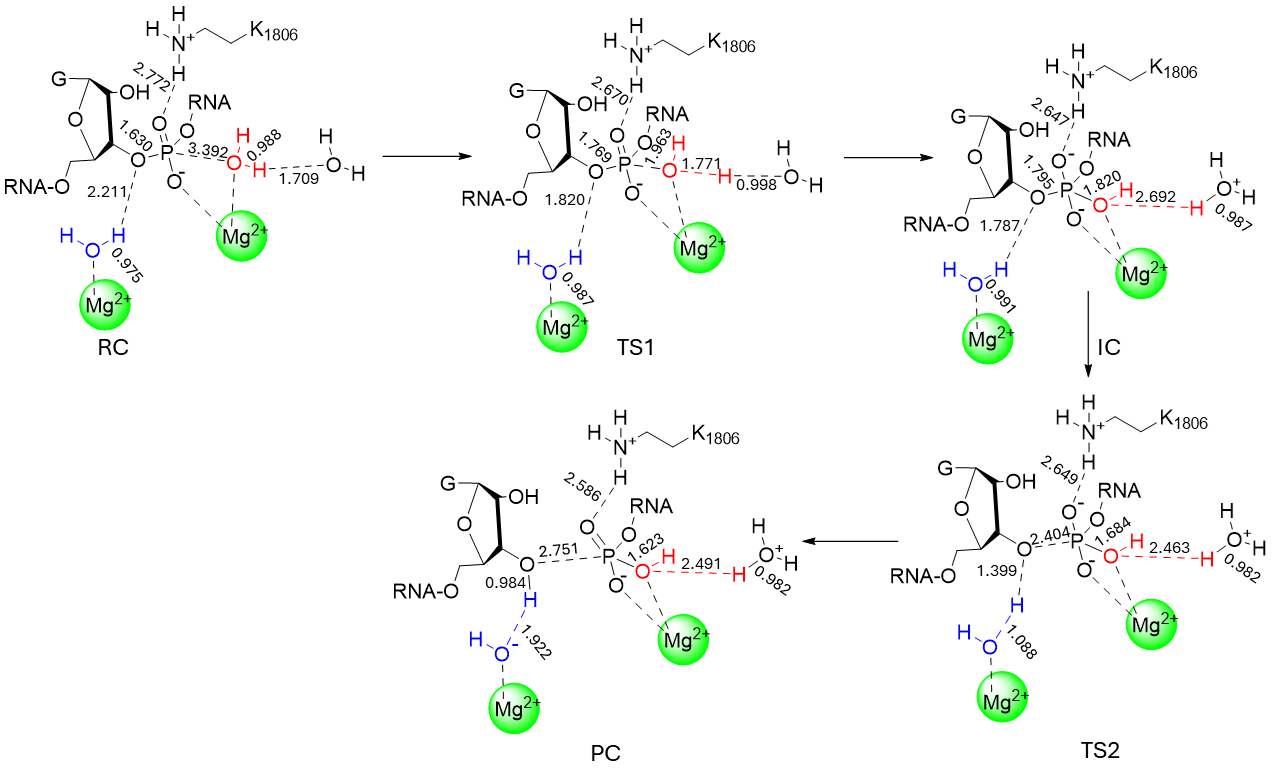


**Figure S11.** ONIOM(M06-2X/6-311+G(2df,p):AMBERff14SB)//ONIOM(M06-2X/6-31G(d,p):AMBERff14SB) calculated Dicer catalytic mechanism involving a water nucleophile obtained using the RIIIb model. Distances reported in Å. Water molecules coordinated to Mg^2+^ ions that do not participate in the reaction are omitted for clarity.


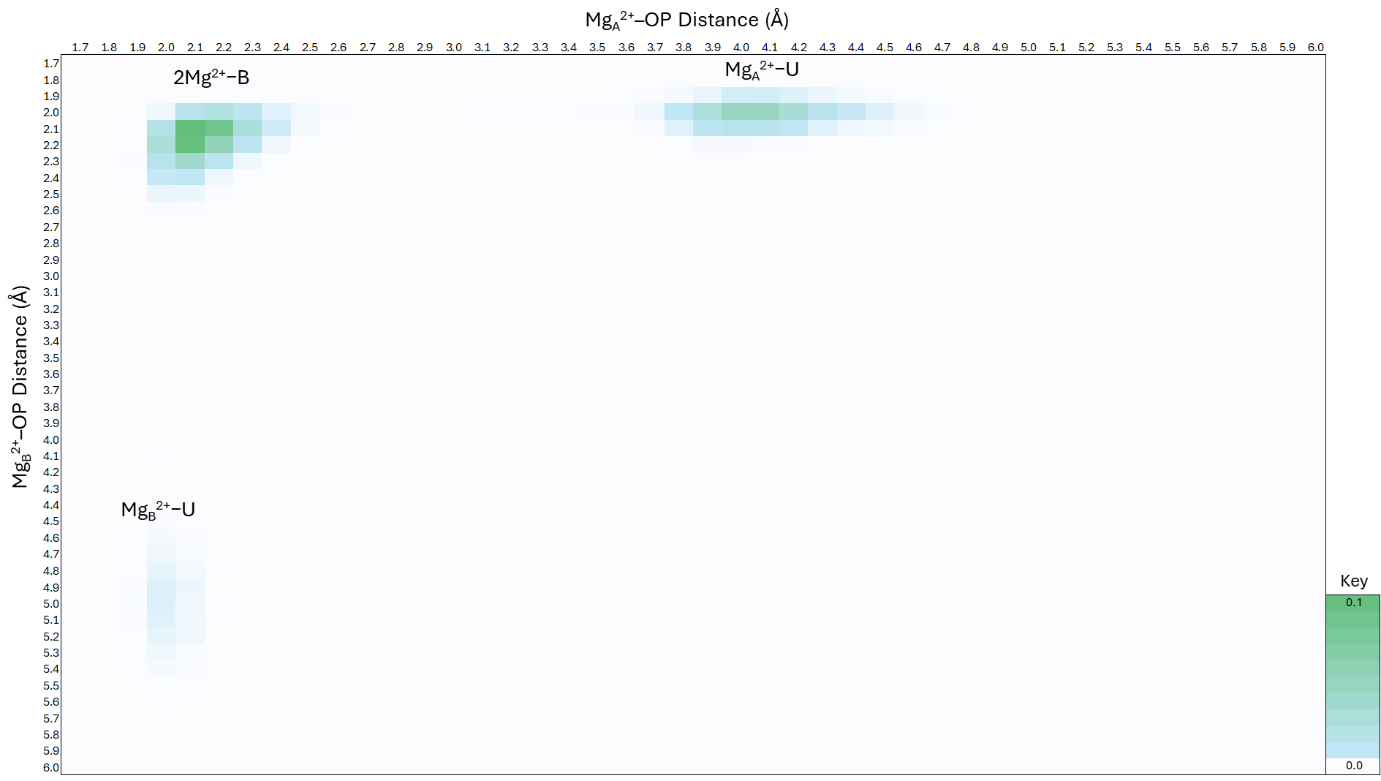


**Figure S12.** Histogram of the fractional occupancies of the distance (Å) between Mg_A_^2+^ or Mg_B_^2+^ and the substrate non-bridging phosphate oxygen over the MD simulations for the aaRIII model. MD representative structures of conformations can be found in Figure 7.


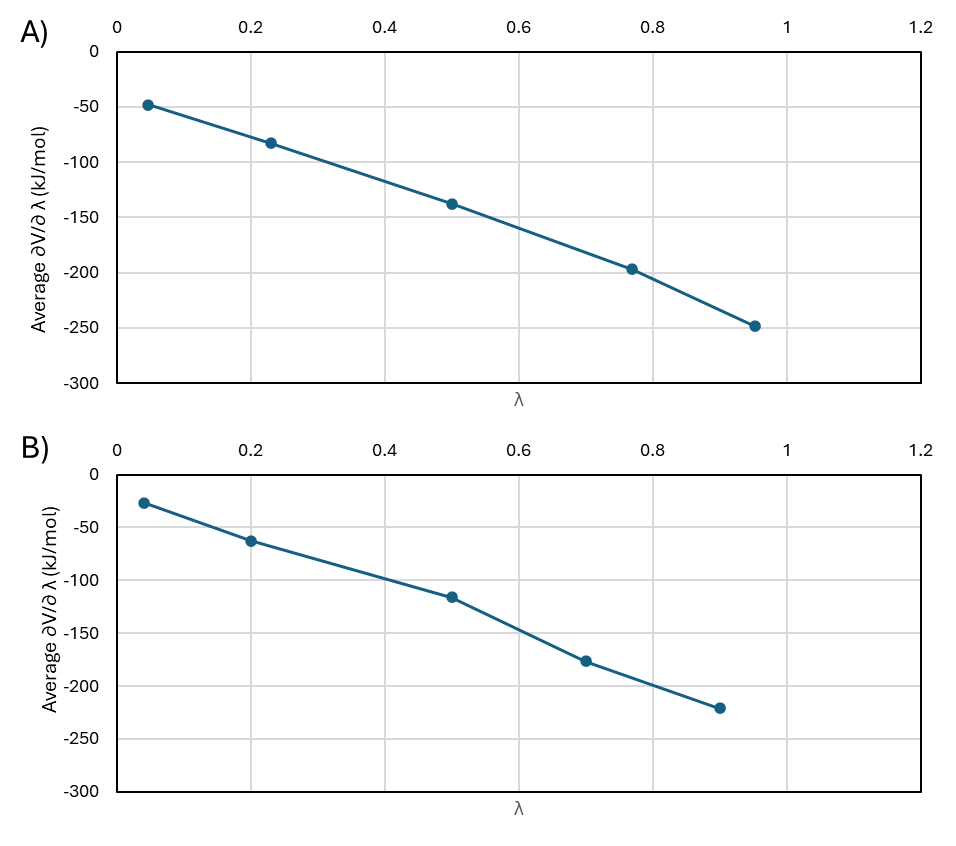


**Figure S13.** Average ∂V/∂λ from thermodynamic integration at different λ values for the alchemical transformation of water into hydroxide in A) Dicer and B) bulk solvent.


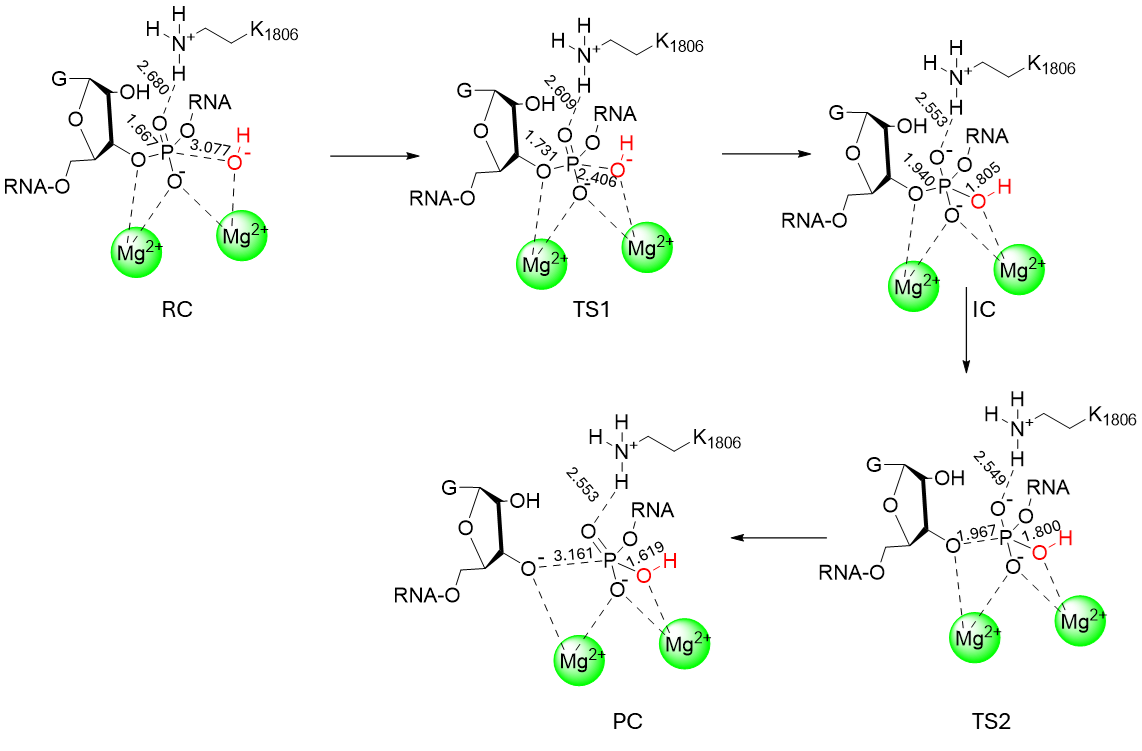


**Figure S14.** ONIOM(M06-2X/6-311+G(2df,p):AMBERff14SB)//ONIOM(M06-2X/6-31G(d,p):AMBERff14SB) calculated Dicer catalytic mechanism involving a hydroxide nucleophile obtained using the aaRIII model. Distances reported in Å.


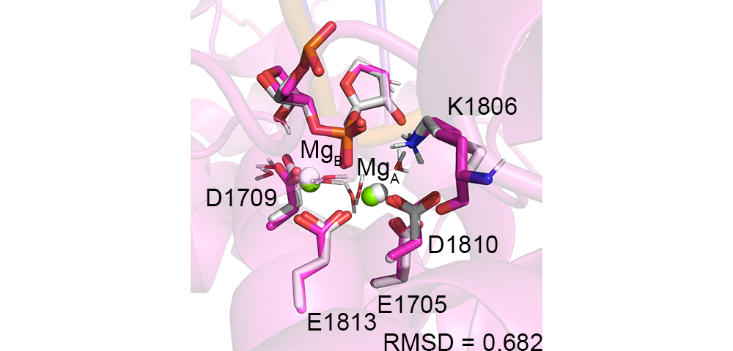


**Figure S15.** Overlay of MD representative structures of the active (2Mg^2+^−B) conformation of the Dicer aaRIII model containing a water (magenta) and hydroxide (white) nucleophile coordinated to Mg_A_^2+^. RMSD is reported in Å.


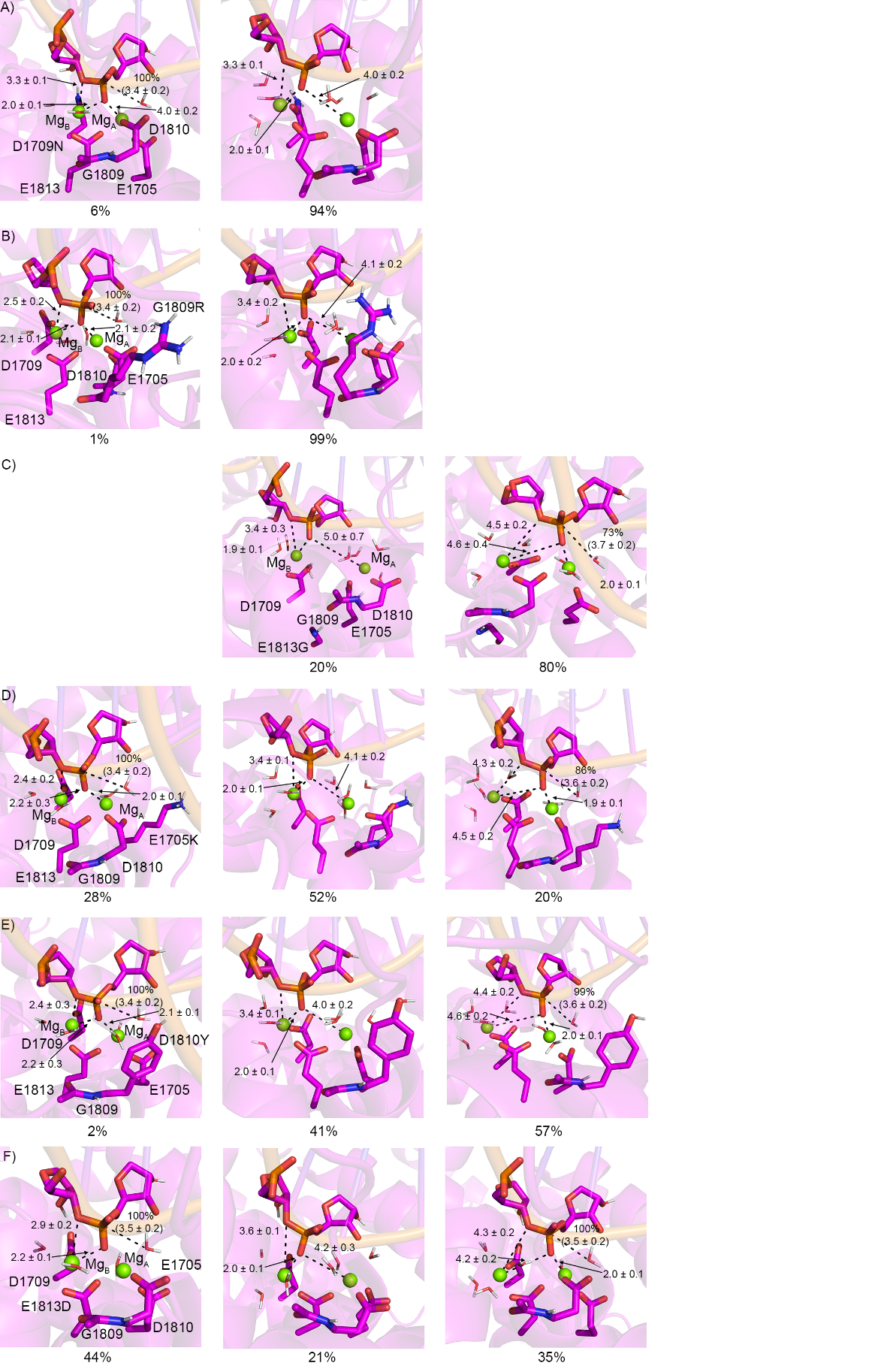


**Figure S16.** MD representative structures for the 2Mg^2+^−B (left), Mg_A_^2+^−U (middle), and Mg_B_^2+^−U (right) conformations for the A) D1709N, B) G1809R, C) E1813G, D) E1705K, E) D1810Y, and F) E1813D Dicer mutants.

**Table S1.** RESP charges for a hydroxide ion calculated at the HF/6-31G(d) level of theory.

| Atom Name | Charge |
| --- | --- |
| O | –1.2049 |
| H1 | 0.2049 |
| H2 | 0.0000 |

**Table S2.** Relative energies of key stationary points for the RIIIb and aaRIII Dicer models.

| Structure | RC | TS1 | IC | TS2 | PC |
| --- | --- | --- | --- | --- | --- |
| RIIIb Uncorrected^a^ | 0.0 | 126.1 | 124.1 | 154.8 | 69.8 |
| RIIIb Corrected^b^ | 0.0 | 253.4 | 269.5 | 293.1 | 221.3 |
| aaRIII Uncorrected  (Water Nucleophile)^a^ | 0.0 | 62.2 | 61.4 | 77.6 | 74.5 |
| aaRIII Corrected  (Water Nucleophile)^b^ | 0.0 | 146.5 | 168.8 | 181.8 | 158.3 |
| aaRIII Uncorrected (Hydroxide Nucleophile)^a^ | 0.0 | 15.1 | –12.8 | –12.8 | –90.1 |
| aaRIII Corrected (Hydroxide Nucleophile)^b^ | 0.0 | 39.2 | 36.8 | 45.0 | 28.9 |

^a^Energies were calculated using ONIOM(M06-2X/6-31G(d,p):AMBERff14SB) with the ME scheme. ^b^Energies were calculated using ONIOM(M06-2X/6-311+G(2df,p):AMBERff14SB)//ONIOM(M06-2X/6-31G(d,p):AMBERff14SB). The EE scheme was used for single-point calculations and a Gibbs energy correction was included.

**Scheme S1.** Thermodynamic cycle used to calculate the ΔΔG_Binding_ for replacing water with hydroxide in the Dicer active site.


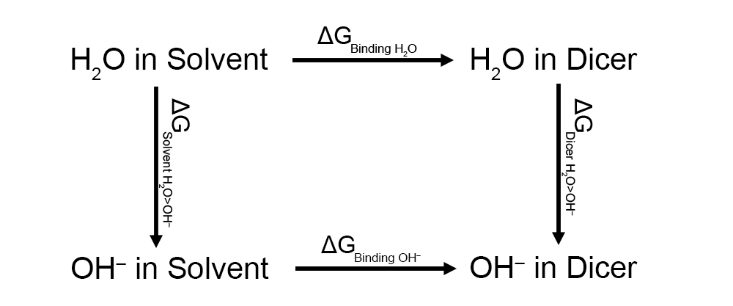


**Equation S1.**

$\Delta G=-k_{B}T ln(\frac{V_{f}}{V_{i}})$
